# A model to simulate human cardio-respiratory responses to airway obstruction

**DOI:** 10.3389/fphys.2025.1699315

**Published:** 2025-11-28

**Authors:** Xin Jin, Varghese Kurian, Kathy L. Ryan, Anders Wallqvist, Jaques Reifman, Sridevi Nagaraja

**Affiliations:** 1 Department of Defense Biotechnology High Performance Computing Software Applications Institute, Defense Health Agency Research and Development, Medical Research and Development Command, Fort Detrick, MD, United States; 2 The Henry M. Jackson Foundation for the Advancement of Military Medicine, Inc., Bethesda, MD, United States; 3 United States Army Institute of Surgical Research, San Antonio, TX, United States

**Keywords:** cardio-respiratory system, mathematical model, airway obstruction, respiratory control, respiratory mechanics

## Abstract

Airway obstruction is the second leading cause of potentially survivable death on the battlefield. Managing airway obstruction resulting from severe traumatic injuries to the head and neck, which can distort the airway anatomy, poses significant challenges to combat medics. The medic’s ability to make quick and effective interventions to secure the airway in austere, tactical environments is also highly dependent on their training and experience as well as the availability of advanced medical equipment. Artificial intelligence (AI) algorithms can help augment the competency and capability of medics to care for combat casualties by enhancing their training, assessing their skills, and helping identify the most appropriate medical interventions in real time that are likely to result in desired clinical outcomes. However, the training and assessment of AI algorithms require massive amounts of real-world, vital-sign data. Because such data are not currently available for casualties with airway obstruction, an alternative approach is to rely on relevant synthetic data generated by high-fidelity computational physiological models. Here, by adding new respiratory control and respiratory mechanics components, we extended our previously developed and validated human cardio-respiratory (CR) model for representing hemorrhagic injury to account for the physiological effects of airway obstruction on vital signs. We calibrated and validated the extended CR model using data from six human studies and two pig studies, which reported vital-sign changes in response to airway obstruction, changes in arterial oxygen (O_2_) and carbon dioxide (CO_2_) pressure, changes in the fraction of inspired O_2_ and CO_2_, and hemorrhage followed by ventilation changes. On average, the extended CR model achieved good prediction accuracy, with root mean square errors of 1.3 L/min for minute ventilation, 1.6 breaths/min for respiratory rate, 4.5% for oxygen saturation, 3.5 mmHg for end-tidal CO_2_, 11.6 mmHg for systolic blood pressure, 7.8 mmHg for mean arterial pressure, and 9.8 beats/min for heart rate. With this enhancement, the extended CR model can now be used to generate realistic synthetic trauma datasets for the two leading causes of potentially survivable battlefield deaths, hemorrhagic injury and airway obstruction, and help develop AI decision-support tools for combat medics.

## 1 Introduction

Battlefield injuries involving penetrating head and neck trauma are becoming increasingly common in current conflicts involving Israeli and Ukrainian forces, with such injury patterns accounting for up to 25% of combat casualties (Lurin et al., 2025; Prysiazhniuk et al., 2025; Tsur et al., 2025). These injuries often result in airway obstruction and massive hemorrhage, with the former usually attributed as the primary cause of death (Breeze and Bryant, 2009; Gibbons and Breeze, 2011; Krausz et al., 2015). In previous conflicts, hemorrhage and airway obstruction together accounted for 99% of the potentially survivable battlefield deaths (Eastridge et al., 2012; Eastridge et al., 2019). Of note, when they occur simultaneously, the U.S. Department of Defense (DoD) Tactical Combat Casualty Care guidelines recommend treating airway obstruction first, before hemorrhage (except in cases of massive blood loss) (Deaton et al., 2021). The diagnosis and emergency treatment of airway obstruction are further complicated by concomitant injuries to the cervical arteries and veins, and failed attempts to perform cricothyroidotomy in the field can increase the risk of recurrent hemorrhage (Mabry et al., 2010; Eastridge et al., 2012). While advances in medical equipment, such as the extraglottic device to treat airway obstruction on the battlefield, can improve clinical outcomes (Hessert and Bennett, 2013; April et al., 2023), it is unlikely that such equipment will be available in future large-scale combat operations (LSCO) against near-peer adversaries, because medical assets will be limited and required to be in near-constant motion (Epstein et al., 2023; United States Army Medical Center of Excellence Lessons Learned Branch, 2025). Under such conditions, enhancing the competency and capability of medics, including the ability to identify appropriate interventions and perform complex medical procedures, could considerably impact the clinical outcome of combat casualties.

Recent advances in artificial intelligence (AI) and machine learning (ML) offer promising technological solutions to augment the competency of combat medics in resource-constrained environments with compromised reach-back capabilities. AI- and ML-based decision-support systems have been proposed to help monitor vital signs of trauma casualties, detect their deterioration, and recommend interventions at or near the point of injury (Jin et al., 2017; Jin et al., 2018; Fernandes et al., 2020; Dolan et al., 2021; Maurer et al., 2021; Peng et al., 2023; Stallings et al., 2023; Jin et al., 2024). However, the training and assessment of AI algorithms for combat casualty care, especially for complex injuries involving both hemorrhage and airway obstruction, face substantial data limitations. First, it is not practical to collect sufficiently large datasets from humans for these injuries. Moreover, large-animal injury models, which may not fully replicate human responses to complex polytrauma, are also limited due to their small sample sizes (Sondeen et al., 2011; Ross et al., 2014; Ziebart et al., 2015; Gerling et al., 2023). Importantly, clinical or experimental studies that capture vital-sign changes when airway obstruction and hemorrhage occur simultaneously are not currently available. To overcome these challenges, synthetic trauma data generated by validated physiological computational models can serve as an effective alternative. Using computational models, we can simulate hundreds of virtual humans and test numerous injury and intervention scenarios to identify optimal treatment strategies without the ethical and practical constraints associated with obtaining sufficient real-world data (Jin et al., 2024). We can then use these diverse datasets for training and assessing AI algorithms.

Over the past 2 decades, computational models of the cardiovascular and respiratory systems have been developed to simulate changes in vital signs resulting from hemorrhagic injury and respiratory perturbations (Chiari et al., 1997; Albanese et al., 2016; Bighamian et al., 2017; Bray et al., 2019). Most small to medium-sized models (with the number of parameters on the order of 10–100) often omit key physiological components, such as airway mechanics or interstitial fluid compartments, limiting their ability to simulate the effect of airway obstruction or hemorrhage on the cardiovascular and respiratory responses (Chiari et al., 1997; Fink et al., 2004; Longobardo et al., 2008; Ellwein et al., 2013; Albanese et al., 2016; Cheng et al., 2016; Karavaev et al., 2016; Trenhago et al., 2016; Bighamian et al., 2017). In contrast, some of the large models, which include thousands of parameters and simulate intertwined interactions across multiple organ systems, are too complex to calibrate and to be seamlessly integrated into AI and ML algorithms (Hester et al., 2011; Bray et al., 2019). Importantly, none of the existing models has been quantitatively validated against experimental data involving airway obstruction.

Recently, our U.S. DoD team developed and validated a human physiological model [the cardio-respiratory (CR) model] to capture the essential cardiovascular and respiratory responses to hemorrhage, fluid resuscitation, and ketamine administration (Jin et al., 2023; Kurian et al., 2025). We validated the CR model using both human and animal data and demonstrated its ability to predict the temporal dynamics of vital signs, including oxygen saturation (SpO_2_), end-tidal carbon dioxide (ETCO_2_), mean arterial pressure (MAP), heart rate (HR), and cardiac output (CO), to reflect hemorrhagic injury, fluid resuscitation, and administration of pain medication. In addition, we utilized this model to generate synthetic data for developing AI algorithms to optimize fluid-resuscitation strategies (Jin et al., 2024).

In this study, we extended the CR model to incorporate the physiological effects of airway obstruction on vital signs, including systolic blood pressure (SBP), diastolic blood pressure (DBP), minute ventilation (MV), and respiratory rate (RR), in addition to those mentioned above. Specifically, we first integrated new components representing respiratory control and respiratory mechanics within the original CR model. We then calibrated and validated the extended model using data from six human studies and two animal studies involving *1*) changes in arterial oxygen (PaO_2_) and carbon dioxide pressures (PaCO_2_), *2*) changes in fractions of inspired oxygen (FiO_2_) and carbon dioxide (FiCO_2_), *3*) airway obstruction, and *4*) hemorrhage followed by ventilation changes. We hypothesized that, in addition to hemorrhagic injuries, the extended CR model will allow us to reliably predict changes in vital signs resulting from airway obstruction.

## 2 Materials and methods

### 2.1 Computational model

**FIGURE 1:**
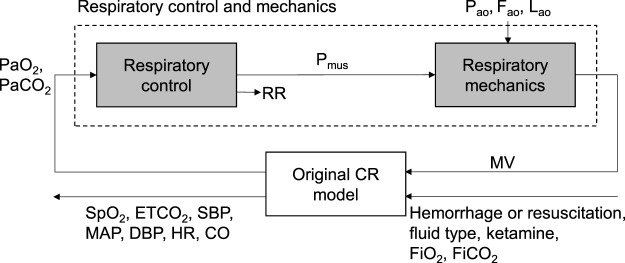
Schematic showing the extended cardio-respiratory (CR) model, with the shaded boxes indicating the model extensions. CO, cardiac output; DBP, diastolic blood pressure; ETCO_2_, end-tidal carbon dioxide; F_ao_, fraction of airway obstruction; FiCO_2_, fraction of inspired carbon dioxide; FiO_2_, fraction of inspired oxygen; HR, heart rate; L_ao_, location of airway obstruction; MAP, mean arterial pressure; MV, minute ventilation; PaCO_2_, arterial carbon dioxide pressure; P_ao_, airway opening pressure; PaO_2_, arterial oxygen pressure; P_mus_, respiratory muscle pressure; RR, respiratory rate; SBP, systolic blood pressure; SpO_2_, oxygen saturation.

We previously developed the CR model, which captures the vital-sign responses to hemorrhage, fluid resuscitation, and ketamine administration (Jin et al., 2023; Kurian et al., 2025). The CR model represents the cardiovascular and respiratory systems, along with their regulatory mechanisms, using a set of 122 ordinary differential and algebraic equations and 143 parameters. Here, we extended the model to account for the effects of airway obstruction on the vital signs, including SpO_2_, ETCO_2_, SBP, MAP, DBP, HR, MV, RR, and CO. To this end, we added two new modules to the CR model: the respiratory control component and the respiratory mechanics component (Figure 1, shaded boxes). Given PaO_2_ and PaCO_2_, the respiratory control component determines the resulting RR and the respiratory muscle pressure (P_mus_). The P_mus_, the airway opening pressure (P_ao_), and the fraction (F_ao_) and location of airway obstruction (L_ao_) serve as inputs to the respiratory mechanics component, which then computes the resulting MV. For a detailed description and implementation of the original CR model formulation, we refer the reader to Jin et al. (2023) and Kurian et al. (2025).

#### 2.1.1 Respiratory control component

**FIGURE 2:**
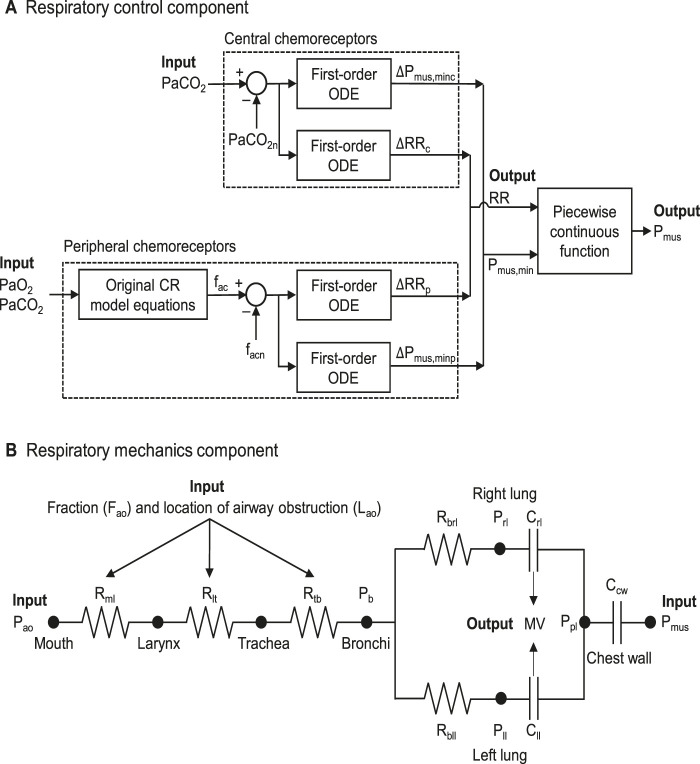
**(A)** Structure of the respiratory control component, which represents the effects of the central and peripheral chemoreceptors on the respiratory muscle pressure (P_mus_). **(B)** Structure of the respiratory mechanics component, which models the airflow dynamics through the mouth, larynx, trachea, bronchi, left lung, right lung, and chest wall. C_cw_, compliance of the chest wall; C_ll_, compliance of the left lung; CR, cardio-respiratory; C_rl_, compliance of the right lung; Δ, delta; f_ac_, activity of peripheral chemoreceptors; f_acn_, nominal activity of peripheral chemoreceptors; MV, minute ventilation; ODE, ordinary differential equation; PaCO_2_, arterial carbon dioxide pressure; PaCO_2n_, nominal value of the arterial carbon dioxide pressure; P_ao_, airway opening pressure; PaO_2_, arterial oxygen pressure; P_b_, bronchial pressure; P_ll_, pressure of the left lung; P_mus,min_, minimum respiratory muscle pressure; ΔP_mus,minc_ and ΔP_mus,minp_, changes in P_mus,min_ caused by central and peripheral chemoreceptors, respectively; P_pl_, pleural pressure; P_rl_, pressure of the right lung; R_bll_, resistance from the bronchi to the left lung; R_brl_, resistance from the bronchi to the right lung; R_lt_, resistance from the larynx to the trachea; R_ml_, resistance from the mouth to the larynx; RR, respiratory rate; ΔRR_c_ and Δ RR_p_, changes in RR caused by central and peripheral chemoreceptors, respectively; R_tb_, resistance from the trachea to the bronchi.

Central chemoreceptors located in the brainstem and peripheral chemoreceptors located in the carotid and aortic arteries play a crucial role in respiration control. These chemoreceptors detect changes in PaO_2_ and PaCO_2_ and respond by regulating the RR and MV (Gourine, 2005). We used the model developed by Albanese et al. to describe the effects of PaO_2_ and PaCO_2_ on RR and P_mus_ (Figure 2A) (Albanese et al., 2016). Because the central chemoreceptors primarily respond to changes in PaCO_2_, in Equations 1, 2 we described their effects on RR and the minimum respiratory muscle pressure (P_mus,min_) by the difference between the current PaCO_2_ and its nominal value (PaCO_2n_), as follows:

$${\Delta \dot{RR}}_{c}+\left(\tau_{\text{cR}}{\;\times \;\Delta \text{RR}}_{c}\right)=G_{\text{cR}}\times \left({\text{PaCO}}_{2\;}-{\text{PaCO}}_{2n}\right)$$ (1)

$$\Delta {\dot{P}}_{\text{mus},\text{minc}}+\left(\tau_{\text{cP}}{\;\times \;\Delta P}_{\text{mus},\text{minc}}\right)=G_{\text{cP}}\times \left({\text{PaCO}}_{2\;}-{\text{PaCO}}_{2n}\right)$$ (2)

where ${\Delta \text{RR}}_{c}$ and ${\Delta P}_{\text{mus},\text{minc}}$ represent changes in RR and P_mus,min_, respectively, caused by the central chemoreceptors, $\tau_{\text{cR}}$ and $\tau_{\text{cP}}$ denote time constants, and $G_{\text{cR}}$ and $G_{\text{cP}}$ represent gain factors. The subscript c denotes central chemoreceptors, R represents RR, and P represents P_mus,min_. The dot “⋅” represents the rate of change of the corresponding variable.

The peripheral chemoreceptors respond to changes in both PaO_2_ and PaCO_2_ (Albanese et al., 2016). In their model, Albanese et al. used a set of 14 equations to compute the activity of peripheral chemoreceptors (f_ac_) as a function of PaO_2_ and PaCO_2_. However, the CR model already includes equations to compute f_ac_, which are similar to those reported by Albanese et al. Therefore, we directly used the f_ac_ values computed in the original CR model to quantify the effects of peripheral chemoreceptors on RR and P_mus,min_ based on the difference between the current f_ac_ and its nominal value (f_acn_), as follows in Equations 3, 4:

$${\Delta \dot{RR}}_{p}+\left(\tau_{\mathrm{pR}}{\;\times \;\Delta \text{RR}}_{p}\right)=G_{\mathrm{pR}}\times \left(f_{\text{ac}}{\;-\;f}_{\text{acn}}\right)$$ (3)

$$\Delta {\dot{P}}_{\text{mus},\text{minp}}+\left(\tau_{\text{pP}}{\;\times \;\Delta P}_{\text{mus},\text{minp}}\right)=G_{\text{pP}}\times \left(f_{\text{ac}}{\;-\;f}_{\text{acn}}\right)$$ (4)

where ${\Delta \text{RR}}_{p}$ and ${\Delta P}_{\text{mus},\text{minp}}$ represent changes in RR and P_mus,min_, respectively, caused by the peripheral chemoreceptors, $\tau_{\mathrm{pR}}$ and $\tau_{\text{pP}}$ denote time constants, and $G_{\mathrm{pR}}$ and $G_{\text{pP}}$ represent gain factors. The subscript p denotes peripheral chemoreceptors, R represents RR, and P represents P_mus,min_. The dot “⋅” represents the rate of change of the corresponding variable.

To compute the final value for RR and P_mus,min_, in Equations 5, 6 below, we added their initial values ( ${\text{RR}}_{o}$ and P_mus, mino_, respectively) to the changes induced in them by the central and peripheral chemoreceptors computed in Equations 3, 4 above, as follows:

$${\text{RR}=\text{RR}}_{o}{\;+\;\Delta \text{RR}}_{c}+{\Delta \text{RR}}_{p}$$ (5)

$$P_{\text{mus},\min}{\;=\;P}_{\text{mus},\text{mino}}{\;+\;\Delta P}_{\text{mus},\text{minc}}+{\Delta P}_{\text{mus},\text{minp}}$$ (6)

Finally, at the start of each respiratory cycle, we generated a time-varying profile of P_mus_ over a single cycle (including both inspiration and expiration) based on the current values of RR and P_mus,min_, using a piecewise continuous function, as described in Albanese et al. (2016). We estimated the duration of each respiratory cycle (T) as 1/RR and the inspiration time (T_i_) as 0.375T. During inspiration (0 to T_i_), P_mus_ decreased from 0 to P_mus,min_, which we characterized by a parabolic function. During expiration (T_i_ to T), P_mus_ increased back to its initial value, which we characterized by an exponential function. Equation 7 shows the entire P_mus_ profile, as follows:

$$P_{\text{mus}}\left(t\right)=\left\{\begin{array}{l} \left({4P}_{\text{mus},\min}\times \text{RR}\times t\right)-\left({4P}_{\text{mus},\min}{\;\times \;\text{RR}}^{2\;}{\;\times \;t}^{2}\right)\;t\in \left[0,T_{i}\right.)\\ \left[\left(e^{\left(3\;-\;8\text{RR}\times t\right)}\;-e^{-5}\right)/\left(1\;-\;e^{-5}\right)\right]\;{\;\times \;P}_{\text{mus},\min}\;t\in \left(T_{i},T\right] \end{array}\right.$$ (7)

We used the P_mus_ profile as one of the inputs to the respiratory mechanics component. Please see Supplementary Table S1 in the Supplementary Material for a list of all parameter values and their definitions.

#### 2.1.2 Respiratory mechanics component

To represent the respiratory mechanics component, we adapted the model developed by Albanese et al. (2016), with P_mus_, P_ao_, F_ao_, and L_ao_ serving as inputs to predict MV as the output (Figure 1). In particular, we used their equations and parameter values with three modifications. First, the original model did not specifically account for airway obstruction. To incorporate this injury, we added an equation representing obstructions at three locations involving both the upper and lower airways: from the mouth to the larynx (mL), from the larynx to the trachea (lt), and from the trachea to the bronchi (tb) (Figure 2B). Following Poiseuille’s law, which relates airway resistance to the cross-sectional area of the airway, in Equation 8 we modeled different obstruction levels by increasing airway resistance R_i_, with i = ml, lt, or tb as follows (Mecklenburgh and Mapleson, 1998):

$$R_{i}=\frac{R_{\text{io}}}{{\left(1\;-\;F_{\text{ao}}\right)}^{2}}$$ (8)

where R_io_ denotes the nominal value of R_i_.

Second, in their original model, Albanese et al. defined individual compliance values for the larynx, trachea, bronchi, lungs, and chest wall. However, we found that the compliance for the larynx, trachea, and bronchi contributed negligibly to the overall respiratory mechanics. Therefore, in the modified mass-balance Equations 9−12, we only considered the individual compliances for the lungs and chest wall. Third, their model represented the lungs as a single compartment. We modified this formulation by representing the left lung and the right lung as two separate compartments. This modification was necessary to simulate unilateral lung injuries, such as lung collapse. To this end, we assumed that the lung compliance was proportional to the lung volume (Benito et al., 1985), and that lung resistance was inversely proportional to lung volume (Lutfi, 2017). Given that the left and right lungs account for approximately 45% and 55%, respectively, of the total lung volume, we applied the same ratio to calculate the corresponding parameters (Szpinda et al., 2014). Specifically, we defined the unstressed volumes of the left lung (V_llu_) and right lung (V_rlu_) and their compliances (C_ll_ and C_rl_) to be 45% and 55%, respectively, of the total lung volume and compliance reported in their model. In addition, we defined the resistances from the bronchi to the left lung (R_bll_) and to the right lung (R_brl_) as 222% and 181%, respectively, of the resistance from the bronchi to the lungs provided in their model. Finally, we duplicated the original mass-balance and volume-change equations for each lung, resulting in Equations 11−14. Based on the second and third modifications, we revised the airflow-dynamics equations by enforcing mass balances of air across all the elements in the respiratory mechanics component, as follows:

$$\frac{P_{\text{ao}\;}-P_{b}}{R_{\text{ml}\;}+R_{\text{lt}\;}+R_{\text{tb}\;}}=\left({\dot{P}}_{\text{pl}\;}-{\dot{P}}_{\text{mus}}\right)\times C_{\text{cw}}$$ (9)

$$\frac{P_{\text{ao}\;}-P_{b\;}}{R_{\text{ml}\;}+R_{\text{lt}\;}+P_{\text{tb}\;}}=\frac{P_{b\;}-P_{\text{ll}\;}}{P_{\text{bll}\;}}+\frac{P_{b\;}-P_{\text{rl}\;}}{P_{\text{brl}\;}}$$ (10)

$$\frac{P_{b\;}-P_{\text{ll}\;}}{P_{\text{bll}\;}}=\left({\dot{P}}_{\text{ll}\;}-{\dot{P}}_{\text{pl}}\right)\times C_{\text{ll}}$$ (11)

$$\frac{P_{b\;}-P_{\text{rl}\;}}{P_{\text{brl}\;}}=\left({\dot{P}}_{\text{rl}\;}-{\dot{P}}_{\text{pl}}\right)\times C_{\text{rl}}$$ (12)

where P_b_ and P_pl_ represent the bronchial pressure and pleural pressure, respectively, C_cw_ denotes the compliance of the chest wall, P_ll_ and P_rl_ denote the pressures of left and right lungs, respectively, and the dot “⋅” represents the rate of change of the corresponding variable.

Next, based on the pressures and compliances of the left and the right lung, we computed their corresponding volumes V_ll_ and V_rl_, respectively, as follows:

$$V_{\text{ll}}=\left[C_{\text{ll}}\times \left(P_{\text{ll}}\;-P_{\text{pl}}\right)\right]+V_{\text{llu}}$$ (13)

$$V_{\text{rl}}=\left[C_{\text{rl}}\times \left(P_{\text{rl}}\;-P_{\text{pl}}\right)\right]+P_{\text{rlu}}$$ (14)

We then added these two volumes together to obtain the total lung volume (V_l_) in Equation 15, as follows:

$$V_{l}=V_{\text{ll}}+V_{\text{rl}}$$ (15)

Finally, at the start of each respiratory cycle, we computed the tidal volume (TV) as the difference between the maximum and minimum V_l_ of the previous respiratory cycle. We then computed MV as the product of TV and RR.

#### 2.1.3 Initialization of the extended CR model

Overall, we added 15 new equations and 20 new parameters to the original CR model. The extended CR model consisted of 137 ordinary differential and algebraic equations and 163 associated parameters (Supplementary Table S1). The model inputs included hemorrhage or resuscitation rates, fluid type, time and dose of ketamine administration, FiO_2_, FiCO_2_, P_ao_, F_ao_, and L_ao_. The model outputs consisted of SpO_2_, ETCO_2_, SBP, MAP, DBP, HR, MV, RR, and CO. Before using the extended CR model to perform a simulation, we must first initialize the inputs to the various model components until they reach a steady state. We started this process by setting the inputs of the original CR model to their nominal values (i.e., FiO_2_ = 21%, FiCO_2_ = 0%, and MV = 6.6 L/min) and setting hemorrhage, fluid resuscitation, and ketamine administration rates to zero (Figure 1). We then provided its outputs (i.e., PaO_2_ and PaCO_2_) as inputs to the newly added respiratory control component to predict RR and P_mus_. Next, we set P_ao_ to zero to represent the atmospheric pressure and F_ao_ to zero to indicate no airway obstruction. These values, together with the predicted P_mus_, were provided to the respiratory mechanics component to predict the MV. Using this updated value for MV, we repeated this process until all outputs of the extended CR model (original CR model plus the two respiratory components) reached a steady state, which took ∼1 s of clock time after initialization.

We solved the model equations using Euler’s method with a time step of 4.17 × 10^−4^ min (Griffiths and Higham, 2010). We performed all simulations using MATLAB 2024b on a desktop computer equipped with an Intel Core i7-14700 processor without a dedicated graphics card. We did not apply any optimization for the central processing units or graphics processing units. However, to improve computational speed, we compiled the MATLAB code into a MATLAB executable file. Using this platform, we simulated multiple experimental scenarios. In general, a simulation of an experimental scenario spanning 1 h (which corresponds to 720 breaths at 12 breaths/min) required ∼1 s of clock time.

### 2.2 Model calibration and validation

We calibrated and validated the extended CR model using the eight experimental studies summarized in Table 1 (Weil et al., 1970; Kronenberg et al., 1972; Reynolds et al., 1972; Reynolds and Milhorn, 1973; Hampson et al., 1990; Dorrington et al., 1997; Blackburn et al., 2019; Blackburn et al., 2021). We selected these studies because their protocols involved a wide range of physiological scenarios, and they reported vital signs predicted by the CR model. Specifically, the physiological scenarios include the following: *1*) PaO_2_ and PaCO_2_ changes in humans [*Studies 1–3* (Weil et al., 1970; Kronenberg et al., 1972; Hampson et al., 1990)], *2*) FiO_2_ and FiCO_2_ changes in humans [*Studies 4–6* (Reynolds et al., 1972; Reynolds and Milhorn, 1973; Dorrington et al., 1997)], *3*) airway obstruction in pigs [*Study 7* (Blackburn et al., 2019)], and *4*) hemorrhage followed by ventilation changes in pigs [*Study 8* (Blackburn et al., 2021)].

**TABLE 1:** Studies used for calibration and validation of the extended cardio-respiratory (CR) model.

Study No.	No. of Subjects	Species	Age (years)	Scenario	Inputs					Outputs	Ref
					PaO_2_ (mmHg)	PaCO_2_ (mmHg)	F_ao_ (%)	P_ao_ (mmHg)	Hemorrhage (% of BV)		
Calibration of the respiratory control and mechanics components based on changes in PaO_2_ and PaCO_2_											
1	4	Human	33	Hypoxia (Subject 1)	36–120	32–37	0	0.0	-	MV	Weil et al. (1970)
			26	Hypoxia (Subject 2)	39–131	38–42	0	0.0	-		
			34	Hypoxia (Subject 3)	48–127	32–39	0	0.0	-		
			35	Hypoxia (Subject 4)	39–141	30–33	0	0.0	-		
Validation of the respiratory control and mechanics components based on changes in PaO_2_ and PaCO_2_											
2	9	Human	22–30	Hypercapnia	40	40–48	0	0.0	-	MV	Kronenberg et al. (1972)
3	8	Human	28–40	Hypocapnic hypoxia	29–100	27–40	0	0.0	-	MV, RR	Hampson et al. (1990)

					FiO_2_ (%)	FiCO_2_ (%)	F_ao_ (%)	P_ao_ (mmHg)	Hemorrhage (% of BV)		
Validation of the extended CR model based on changes in FiO_2_ and FiCO_2_											
4	6	Human	29–57	Hypoxia	10–21	0–2	0	0.0	0	SpO_2_, ETCO_2_, SBP, HR	Dorrington et al. (1997)
5	10	Human	22–29	Hypercapnia	21	3	0	0.0	0	MV, RR	Reynolds et al. (1972)
6	10	Human	22–29	Hypoxia	9	0	0	0.0	0	MV, RR	Reynolds and Milhorn (1973)
Validation of the extended CR model based on airway obstruction											
7	16	Pig	-		21	0	0–100^a^	0.0	0	SpO_2_, ETCO_2_, SBP, HR	Blackburn et al. (2019)
Validation of the extended CR model based on hemorrhage followed by changes in ventilation											
8	50	Pig	-	Spontaneous ventilation	21	0	0	0.0	25	ETCO_2_, MAP, HR	Blackburn et al. (2021)
				Normal ventilation	21	0	0	0.0–0.6	25		
				Hyperventilation	21	0	0	0.0–4.3	25		
				Max hyperventilation	21	0	0	0.0–4.6	25		
				High FiO_2_ ventilation	30	0	0	0.0–0.7	25		

BV, blood volume; ETCO_2_, end-tidal carbon dioxide; F_ao_, fraction of airway obstruction; FiCO_2_, fraction of inspired carbon dioxide; FiO_2_, fraction of inspired oxygen; HR, heart rate; MAP, mean arterial pressure; MV, minute ventilation; PaCO_2_, arterial carbon dioxide pressure; P_ao_, airway opening pressure; PaO_2_, arterial oxygen pressure; RR, respiratory rate; SBP, systolic blood pressure; SpO_2_, oxygen saturation.

^a^ Airway obstruction located from the mouth to the larynx.

From the eight studies, we used *Study 1* to calibrate the newly added respiratory (control and mechanics) components and *Studies 2* and *3* to validate their predictions. *Studies 1–3* reported changes in model outputs (MV and RR) in response to varying levels of model inputs (PaO_2_ and PaCO_2_). We specifically selected *Study 1* to calibrate the respiratory components because this study covered the largest variation of the inputs to the model. Finally, we used *Studies 4–8* to validate the full, extended CR model in Figure 1. We refer the reader to the original articles for additional information (Weil et al., 1970; Kronenberg et al., 1972; Reynolds et al., 1972; Reynolds and Milhorn, 1973; Hampson et al., 1990; Dorrington et al., 1997; Blackburn et al., 2019; Blackburn et al., 2021).

#### 2.2.1 Calibration of the respiratory-control and respiratory-mechanics components

To ensure that the extended CR model accurately captured the vital-sign responses to ventilation perturbations, we calibrated eight of the 20 parameters in the respiratory control and mechanics components, including four time constants ( $\tau_{\text{cR}}$ , $\tau_{\text{cP}}$ , $\tau_{\mathrm{pR}}$ , and $\tau_{\text{pP}}$ ) and four gain factors ( $G_{\text{cR}}$ , $G_{\text{cP}}$ , $G_{\mathrm{pR}}$ , and $G_{\text{pP}}$ ), associated with the central and peripheral chemoreceptors in Equations 1−4. These are the same parameters that Albanese et al. calibrated when they integrated these respiratory components with their own cardio-respiratory model (Albanese et al., 2016). For the remaining parameters, we kept their original values.

To perform the calibration, we used the MV data reported in *Study 1*, where four human subjects were exposed to a wide range of PaO_2_ change (36–141 mmHg) and PaCO_2_ change (30–42 mmHg) (Table 1). We first defined the feasible ranges for the eight parameters as 0.5-fold to 2-fold of their nominal values, as reported by Albanese et al. Next, we employed Latin Hypercube Sampling to generate 10,000 unique parameter sets, where the values of the eight parameters were randomly selected within their specified ranges (Helton and Davis, 2003). Using each parameter set, we simulated each of the four subjects resulting in a total of 40,000 simulations. Because the MV values at baseline varied between the different experiments as well as between the model simulations, we normalized the predicted MV by multiplying it by the ratio of its baseline experimental value to its baseline simulated value. Next, for every simulation, we calculated the root mean square error (RMSE) between the experimental and normalized predicted MV. To identify the parameter values for calibration, we selected the parameter set for which the sum of the RMSEs across the four subjects was the smallest. We defined this final set of parameter values as the model’s “nominal parameter set.”

#### 2.2.2 Validation of the respiratory control and mechanics components

To validate the two newly added components, we compared the MV predictions with experimental data from *Studies 2* and *3* (Table 1), which involved human subjects challenged with changes in PaO_2_ (29–100 mmHg) and PaCO_2_ (27–48 mmHg). To replicate the scenarios in these studies, we used the measured values of PaO_2_ and PaCO_2_ as inputs to the respiratory control component and recorded the predicted values of MV and RR. As in the calibration procedure, we normalized these predictions and compared them with the corresponding experimental data, using two metrics: the RMSE between the normalized predictions and the measured data and the percentage of the normalized predictions that fell within two standard errors of the mean (SEM) of the experimental data. The latter metric allows us to estimate the extent to which the model predictions were indistinguishable from the mean of the experimental data.

#### 2.2.3 Validation of the extended CR model

We validated the extended CR model (after the integration of the respiratory control and mechanics components) by comparing its predictions with experimental data from five existing studies (*Studies 4–8* in Table 1). *Studies 4–6* reported the effects of changes in FiO_2_ (9%–21%) and FiCO_2_ (0%–3%) on six physiological responses (SpO_2_, ETCO_2_, SBP, HR, MV, and RR) in human subjects (Reynolds et al., 1972; Reynolds and Milhorn, 1973; Dorrington et al., 1997). To replicate these scenarios *in silico*, we used the measured values of FiO_2_ and FiCO_2_ as inputs to the extended CR model and recorded the predicted values of the six outputs. Then, we normalized the model predictions, compared them with the corresponding experimental data, and assessed the comparison by computing the same two metrics discussed above, i.e., the RMSE and the percentage of the normalized predictions that fell within 2 SEM of the experimental data.

*Study 7* involved ventilated pigs subjected to both slow and rapid airway-obstruction protocols, which were implemented by gradually tightening a large thoracic hemostatic clamp on the ventilation tube. In the slow protocol, the animals were challenged with 25%, 50%, and 75% obstructions, each maintained for approximately 8 min, followed by 3 min of complete (100%) obstruction (Blackburn et al., 2019). In the rapid protocol, the animals were only challenged with a 100% obstruction for 3 min. This study was conducted at the U.S. Army Institute of Surgical Research in Fort Sam Houston, Texas, for which we had access to the measured raw time-series data for four vital signs (SpO_2_, ETCO_2_, SBP, and HR) for each individual pig. We processed the raw data by applying an ℓ_1_ trend filter with a regularization parameter of 50 (Kim et al., 2009). To replicate the airway-obstruction scenario *in silico*, we used the measured F_ao_ at the airway segment from the mouth to the larynx as the input to the extended CR model and recorded the model predictions for the four vital signs. To validate the model for the 0%, 25%, 50%, and 75% obstruction phases, we compared the normalized model predictions for the four vital signs with the corresponding experimental data at the end of each obstruction level and computed the same two metrics described above. However, for the 100% obstruction phase, because the vital signs changed very rapidly, we defined a 3-min window around the reported release time (from 2 min pre-obstruction to 1 min post-obstruction) and extracted the vital-sign values that exhibited the largest deviation from their corresponding baseline values to represent the response of the 100% obstruction. We used these values to validate the model predictions.

*Study 8* involved 50 pigs divided into five groups, with each group subjected to a controlled 25% blood loss by volume over 20 min, followed by one of five ventilation scenarios for 60 min: *1*) spontaneous ventilation (∼4.0 L/min), *2*) 4.8 L/min MV by Ambu bag, *3*) 9.0 L/min MV by Ambu bag, *4*) 15.0 L/min MV by Ambu bag, or *5*) 4.1 L/min MV with 30% FiO_2_ using a mechanical ventilator (Blackburn et al., 2021). After the controlled-ventilation phase, all animals were returned to spontaneous ventilation for an additional 10 min. To replicate these experimental scenarios *in silico*, we provided three inputs to the extended CR model: hemorrhage rate, FiO_2_, and P_ao_. During spontaneous ventilation, we set P_ao_ to zero to represent the atmospheric pressure. However, because the experimental study did not report the values of P_ao_ during the controlled-ventilation phase, we adjusted it to ensure that the MV predicted by the respiratory mechanics component of the CR model matched the measured MV values used in each of the five groups. Because our model represents human physiology while the experiments were performed on pigs, we normalized both the hemorrhage rate and MV inputs. Specifically, we converted the 25% blood-volume loss in pigs to a human-equivalent hemorrhage by multiplying this percentage by the estimated blood volume of a 70-kg adult (5 L), as previously described (Jin et al., 2023). Next, to obtain human-equivalent MV values, we scaled the reported MV by the ratio of 70 kg to the average pig body weight in the study (45 kg). Finally, to validate the model, we compared the normalized predicted values of ETCO_2_, MAP, and HR across the five groups with their corresponding experimentally measured values and computed the same two metrics discussed above for these three vital signs (the outputs of the CR model).

### 2.3 Sensitivity analysis

To identify the CR model parameters that influenced model outputs the most during airway obstruction, we performed a local sensitivity analysis (Mitrophanov et al., 2007). In total, we analyzed 327 simulations. First, using the nominal parameter values, we simulated the slow airway obstruction protocol described in *Study 7*, which involved a progressive airway obstruction in pigs, including 0%, 25%, 50%, 75%, and 100%. Next, we perturbed each of the 163 parameters, one at a time, by +10% and by −10% of their nominal values and repeated the same simulation 326 times. We calculated the local sensitivities of the model’s *i*th output to changes in the model’s *j*th parameter (of 163 parameters), S_ij_(t), at timepoint t, as follows in Equation 16:

$$S_{\text{ij}}\left(t\right)=\frac{\left[y_{\text{ij}+}\left(t\right)-y_{\text{ij}‐}\left(t\right)\right]}{{0.2\times y}_{i0}\left(t\right)}$$ (16)

where y(t) denotes the model’s output. The subscripts “+” and “–” denote the outputs computed when a given parameter value was increased and decreased, respectively, by 10% of its nominal value. The subscript “0” denotes the output computed using the nominal parameter values. Then, at the end of each simulation, we analyzed the sensitivities of six key outputs, including the four vital signs reported in *Study 7* (SpO_2_, ETCO_2_, SBP, and HR) and two intermediate outputs of the respiratory components (MV and RR), to changes in each of the 163 parameters at five distinct simulation timepoints, i.e., at the end of 0% (baseline), 25%, 50%, 75%, and 100% obstructions. For each of the six outputs, we ranked the absolute values of the 163 sensitivities in descending order to identify the two most sensitive parameters.

## 3 Results

### 3.1 Calibration of the respiratory control and mechanics components

**FIGURE 3:**
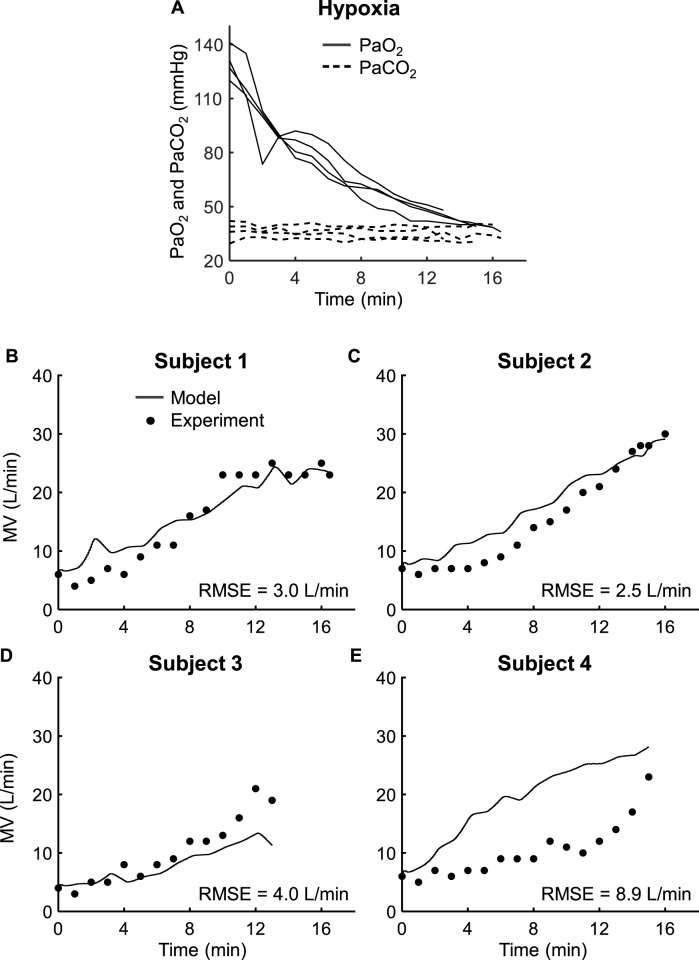
Input profile and model calibration results using the experimental data from *Study 1*. **(A)** Arterial oxygen pressure (PaO_2_, solid lines) and arterial carbon dioxide pressure (PaCO_2_, dashed lines) for four subjects. **(B–E)** Experimental (filled circles) and calibration results (solid lines) for minute ventilation (MV) for each of the four subjects in *Study 1*. RMSE: root mean square error.

We calibrated eight parameters of the respiratory control and mechanics components, including the time constants ( $\tau_{\text{cR}}$ , $\tau_{\text{cP}}$ , $\tau_{\mathrm{pR}}$ , and $\tau_{\text{pP}}$ ) and gain factors ( $G_{\text{cR}}$ , $G_{\text{cP}}$ , $G_{\mathrm{pR}}$ , and $G_{\text{pP}}$ ) of the central and peripheral chemoreceptors, using data from *Study 1*, which reported MV responses of four human subjects to changes in PaO_2_ (36–141 mmHg; Figure 3A, solid lines) and PaCO_2_ (30–42 mmHg; Figure 3A, dashed lines). Figures 3B–E show the calibrated model results for MV (solid lines) and the corresponding experimentally measured data (filled circles) for each of the four subjects. For Subjects 1–3, the model captured the overall trends and showed reasonable agreement with the measurements (Figures 3B–D), with RMSEs for these three subjects ranging between 2.5 and 4.0 L/min (Table 2). For Subject 4, the model overestimated MV (Figure 3E), resulting in a considerably higher RMSE of 8.9 L/min (Table 2).

**TABLE 2:** Root mean square error (RMSE) between the normalized model predictions and the measured experimental data and percentage of the normalized model predictions that fell within two standard errors of the mean of the experimental data for each of the eight studies.

Study No.	MV		RR		SpO_2_		ETCO_2_		MAP or SBP		HR	
	RMSE (L/min)	%	RMSE (breaths/ min)	%	RMSE (%)	%	RMSE (mmHg)	%	RMSE (mmHg)	%	RMSE (beats/ min)	%
*Calibration of the respiratory control and mechanics components*												
1	3.0	-	-	-	-	-	-	-	-	-	-	-
	2.5	-	-	-	-	-	-	-	-	-	-	-
	4.0	-	-	-	-	-	-	-	-	-	-	-
	8.9	-	-	-	-	-	-	-	-	-	-	-
Validation of the respiratory control and mechanics components												
2	13.9	100	-	-	-	-	-	-	-	-	-	-
3	5.8	100	6.4	0	-	-	-	-	-	-	-	-
Validation of the extended cardio-respiratory model												
4	-	-	-	-	3.9	42	2.5	25	6.0^a^	79^a^	9.1	42
5	1.5	47	1.3	95	-	-	-	-	-	-	-	-
6	1.1	58	1.9	100	-	-	-	-	-	-	-	-
7	-	-	-	-	5.0	67	11.6	50	17.1^a^	50^a^	28.2	67
8	-	-	-	-	-	-	1.3	100	6.1	91	5.4	100
	-	-	-	-	-	-	1.9	73	7.0	91	8.8	100
	-	-	-	-	-	-	4.2	36	4.8	100	9.6	100
	-	-	-	-	-	-	1.5	82	8.4	64	3.2	100
	-	-	-	-	-	-	1.6	100	12.6	91	4.4	100

ETCO_2_, end-tidal carbon dioxide; HR, heart rate; MAP, mean arterial pressure; MV, minute ventilation; RR, respiratory rate; SpO_2_, oxygen saturation.

^a^ RMSE for systolic blood pressure.

### 3.2 Validation of the respiratory control and mechanics components

**FIGURE 4:**
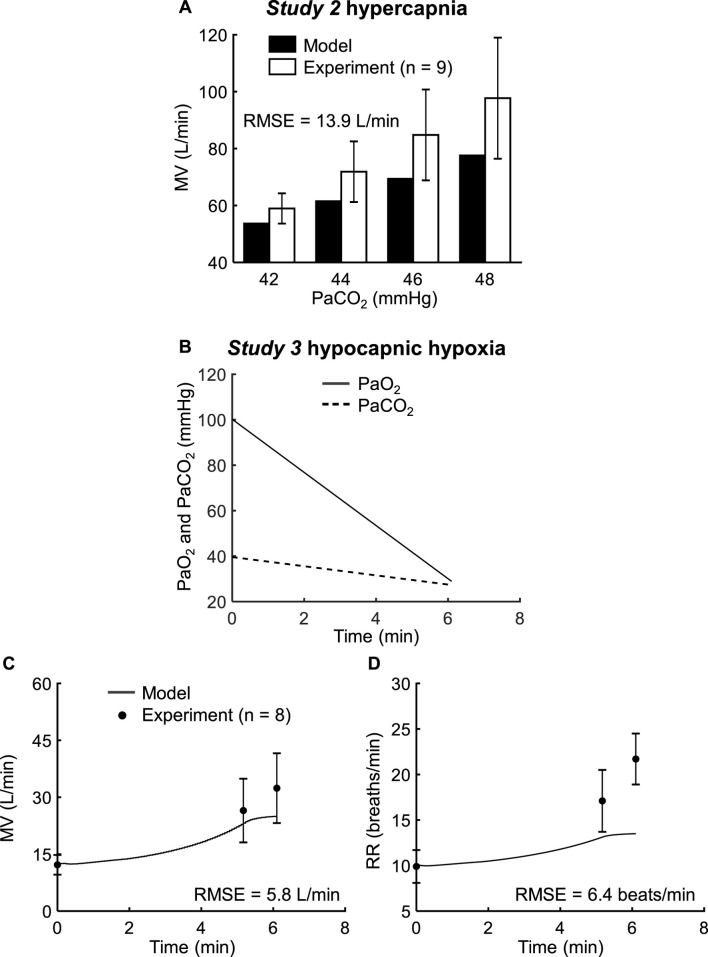
Input profile and model validation results using the experimental data from *Studies 2* and *3*. **(A)** Comparison of the experimental steady-state minute ventilation (MV) data (open bars) from *Study 2* with the corresponding model predictions (solid bars) across different levels of arterial carbon dioxide pressure (PaCO_2_). **(B)** Model inputs for *Study 3* included the arterial oxygen pressure (PaO_2_, solid line) and PaCO_2_ (dashed line). **(C,D)** Experimental (filled circles) and prediction results (solid lines) for MV and respiratory rate (RR) from *Study 3*. The error bars denote two standard errors of the mean. RMSE: root mean square error.

To validate the two respiratory components, we compared the predicted changes in MV and RR with the corresponding experimental measurements from *Studies 2* and *3*. *Study 2* reported changes in MV in response to increases in PaCO_2_ (ranging from 40 to 48 mmHg) for a constant PaO_2_ of 40 mmHg. Figure 4A shows the model predictions of MV after reaching a steady state following each increment of PaCO_2_ (from 40 to 42, 44, 46, and 48 mmHg; solid bars) and the corresponding experimental measurements (open bars). While the model tended to underpredict the experimental data, it yielded an RMSE of 13.9 L/min, with 100% of the predicted values falling within 2 SEM of the experimental data (Figure 4A; Table 2).

*Study 3* reported changes in MV and RR in response to decreases in both PaO_2_ (from 100 to 29 mmHg; Figure 4B, solid line) and PaCO_2_ (from 40 to 27 mmHg; Figure 4B, dashed line). Figures 4C,D show the model predictions (solid lines) for MV and RR, respectively, and the corresponding experimental measurements (filled circles). The predictions for MV showed reasonable agreement with the experimental data, with an RMSE of 5.8 L/min and with 100% of the predictions falling within 2 SEM of the experimental data (Figure 4C; Table 2). However, for RR, we observed considerable differences between the model predictions and the experimental data, with none of the predictions falling within 2 SEM of the experimental data and an RMSE of 6.4 breaths/min (Figure 4D; Table 2).

### 3.3 Validation of the extended CR model

To validate the integrated extended CR model, we compared its predictions of time-course changes in SpO_2_, ETCO_2_, SBP, HR, MV, and RR with the corresponding experimental data from *Studies 4–8*. These studies covered a variety of experimental conditions, including changes in FiO_2_ and FiCO_2_ in humans (*Studies 4–6*), airway obstruction in pigs (*Study 7*), and hemorrhage followed by ventilation changes in pigs (*Study 8*).

**FIGURE 5:**
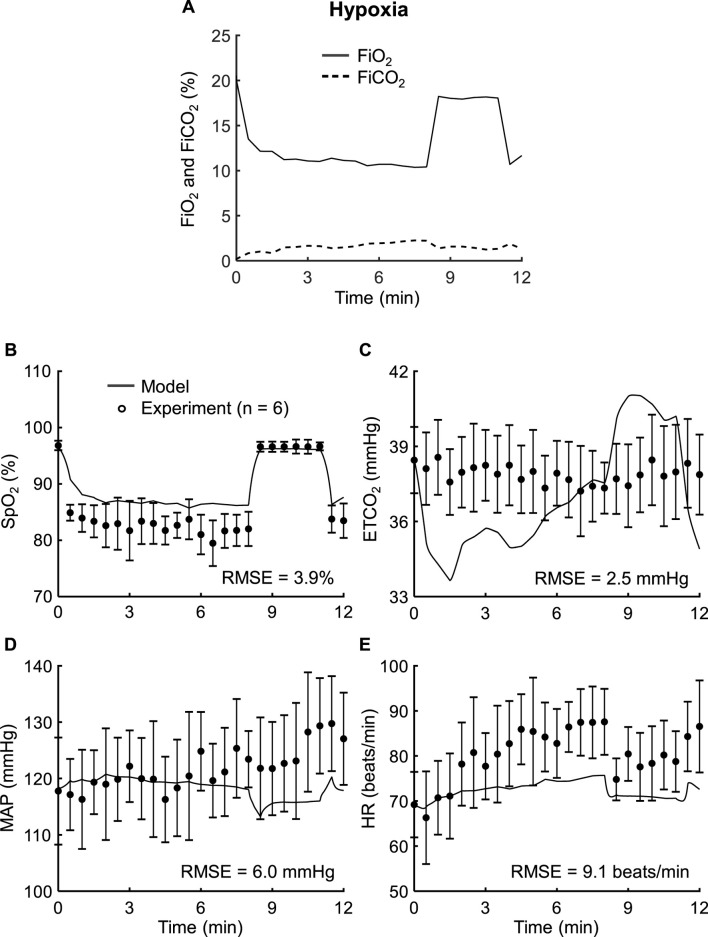
Input profile and model validation results using the experimental data from *Study 4*. **(A)** Model inputs for this study included the fractions of inspired oxygen (FiO_2_, solid line) and carbon dioxide (FiCO_2_, dashed line). **(B–E)** Experimental (filled circles) and prediction results (solid lines) for oxygen saturation (SpO_2_), end-tidal carbon dioxide (ETCO_2_), mean arterial pressure (MAP), and heart rate (HR). The error bars denote two standard errors of the mean. RMSE: root mean square error.

*Study 4* reported vital-sign changes in response to changes in FiO_2_ (10%–21%; Figure 5A, solid line) and FiCO_2_ (0%–2%; Figure 5A, dashed line). Figures 5B–E show the model predictions (solid lines) and the corresponding experimental measurements (filled circles) for SpO_2_, ETCO_2_, MAP, and HR, respectively. The model predictions showed reasonable agreement with the experimentally measured data, with RMSEs of 3.9% for SpO_2_, 6.0 mmHg for MAP, and 9.1 beats/min for HR, with 42%–79% of the predictions falling within 2 SEM of the corresponding experimental data (Table 2). For ETCO_2_, only 25% of the predictions fell within 2 SEM of the experimental data. However, the RMSE (2.5 mmHg) was comparable to the instrument accuracy (Table 2) (Martin-Gill et al., 2024).

**FIGURE 6:**
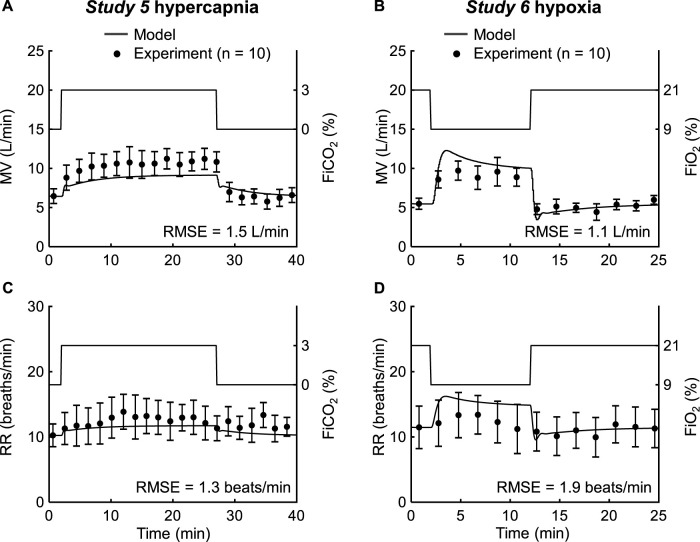
Input profile and model validation results using the experimental data from *Studies 5* and *6*. **(A,B)** Model input profiles on the right y-axis: fraction of inspired carbon dioxide (FiCO_2_, solid line) from *Study 5* and fraction of inspired oxygen (FiO_2_, solid line) from *Study 6*. The left y-axis of these two panels display the corresponding experimental (filled circles) and prediction results (solid lines) for minute ventilation (MV). **(C,D)** Input profiles on the right y-axis: FiCO_2_ (solid line) from *Study 5* and FiO_2_ (solid line) from *Study 6*. The left y-axis of these two panels displays the corresponding experimental (filled circles) and prediction results (solid lines) for respiratory rate (RR). The error bars denote two standard errors of the mean. RMSE: root mean square error.

*Studies 5* and *6* reported changes in MV and RR in response to changes in FiO_2_ (9%–21%; Figures 6B,D, solid lines on the right y-axis) and changes in FiCO_2_ (0%–3%; Figures 6A,C, solid lines on the right y-axis). Figures 6A,C show the model predictions (solid lines) and the corresponding experimental measurements (filled circles) for *Study 5* for MV and RR, respectively. Figures 6B,D show the corresponding results for *Study 6*. In both studies, the model predictions showed good agreement with the experimental data. On average, across the two studies, we obtained RMSEs of 1.3 L/min for MV and 1.6 breaths/min for RR, with 53% of the MV predictions and 98% of the RR predictions falling within 2 SEM of the experimental data (Table 2). The prediction results from *Studies 4–6* demonstrated that the model simulated the cardiovascular and respiratory responses to changes in FiO_2_ and FiCO_2_ across different experimental scenarios.

**FIGURE 7:**
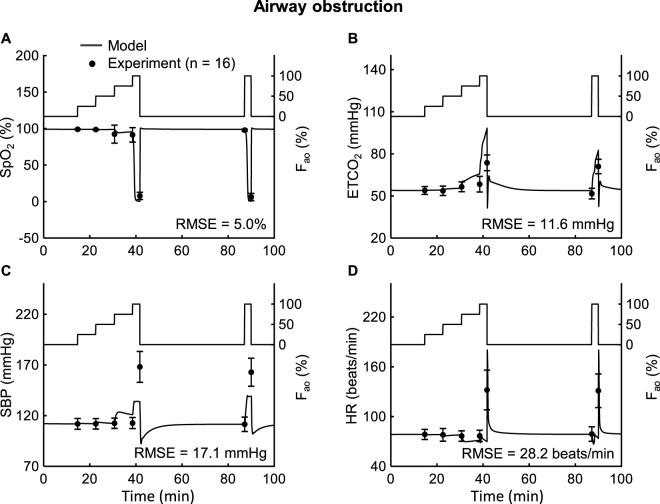
Input profile and model validation results using the experimental data from *Study 7*. **(A–D)** Model input profile on the right y-axis: fraction of airway obstruction (F_ao_, solid lines). The left y-axis of these panels show the experimental (filled circles) and prediction results (solid lines) for oxygen saturation (SpO_2_), end-tidal carbon dioxide (ETCO_2_), systolic blood pressure (SBP), and heart rate (HR). The error bars denote two standard errors of the mean. RMSE: root mean square error.

*Study 7* reported changes in vital signs for airway obstruction ranging from 0% to 100% (Figures 7A–D, solid lines on the right y-axis). Figures 7A–D also show the model predictions (solid lines) and the corresponding experimental data (filled circles) for SpO_2_, ETCO_2_, SBP, and HR. The model yielded a small RMSE of 5.0% for SpO_2_, while the RMSEs for ETCO_2_ (11.6 mmHg), SBP (17.1 mmHg), and HR (28.2 beats/min) were relatively larger (Table 2). The 100% airway obstruction case was responsible for these large discrepancies. In the absence of this extreme case, the model yielded RMSEs of 3.8 mmHg for ETCO_2_, 4.1 mmHg for SBP, and 2.7 beats/min for HR (Figures 7B–D). For the 0%–75% airway obstruction, the model reliably predicted the general trends of each of the four outputs, with 75%–100% of the predictions falling within 2 SEM of the experimental data. Overall, the model demonstrated high fidelity in reproducing the cardiovascular and respiratory responses to airway obstruction up to 75%.

**FIGURE 8:**
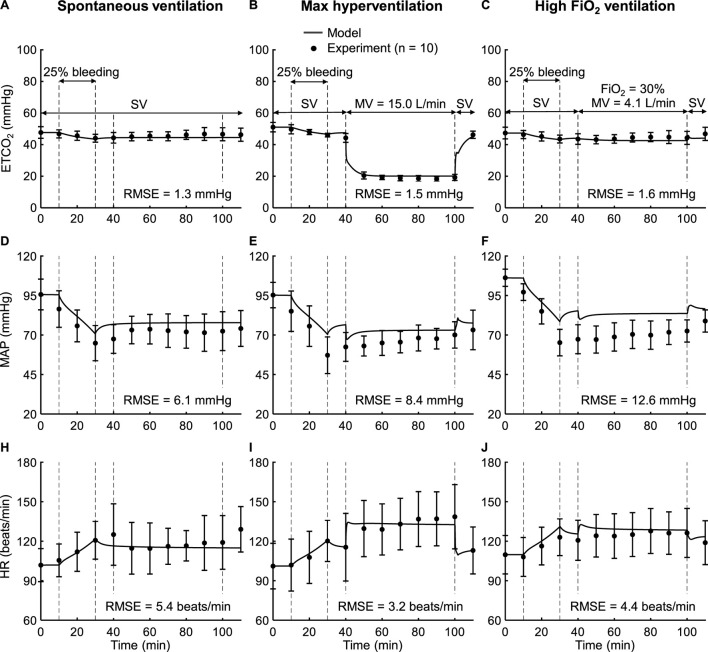
Input profile and model validation results using the experimental data from *Study 8* for the spontaneous ventilation, max hyperventilation, and high FiO_2_ (fraction of inspired oxygen) ventilation scenarios. Experimental (filled circles) and prediction results (solid lines) for **(A–C)** end-tidal carbon dioxide (ETCO_2_), **(D–F)** mean arterial pressure (MAP), and **(H–J)** heart rate (HR). The error bars denote two standard errors of the mean. The timelines at the top of panels **(A–C)** illustrate the experimental scenario, where the subjects underwent hemorrhage (25% of blood volume) followed by ventilation changes. SV indicates that the subjects were on spontaneous ventilation during that period. MV: minute ventilation; RMSE: root mean square error.

*Study 8* reported changes in vital signs for hemorrhage (25% of blood volume) followed by five different ventilation scenarios. Figure 8 shows the model predictions (solid lines) and the corresponding experimental measurements (filled circles) for ETCO_2_, MAP, and HR under three of the five scenarios: spontaneous ventilation, max hyperventilation, and high FiO_2_ ventilation. Supplementary Figure S1 shows the corresponding comparisons for the remaining two scenarios: normal ventilation and hyperventilation. We observed good agreement between the model predictions and the experimental data for all three outputs in each of the five groups. On average, across the five groups, we obtained small RMSEs of 2.1 mmHg for ETCO_2_, 7.8 mmHg for MAP, and 6.3 beats/min for HR (Table 2). Except for ETCO_2_, where only 36% of the model predictions fell within 2 SEM of the experimental data during hyperventilation, at least 64% of the model predictions for the remaining outputs fell within 2 SEM of the measured experimental data (Table 2). Thus, the model reliably captured the cardiovascular and respiratory responses to ventilation changes following a hemorrhagic injury.

### 3.4 Sensitivity analysis

We identified the CR model parameters that had the largest influence on six key outputs (SpO_2_, ETCO_2_, SBP, HR, MV, and RR) by performing a local sensitivity analysis. In Table 3, for the four vital-sign outputs (SpO_2_, ETCO_2_, SBP, and HR), we list the top two parameters (out of 163) with the highest and second highest absolute sensitivities at five simulation timepoints representing 0%, 25%, 50%, 75%, and 100% airway obstruction. For 0%–75% obstruction, SpO_2_ was most sensitive to the two parameters of the O_2_-hemoglobin dissociation sigmoidal function, γ and P_50_ (described in Supplementary Table S1 in the Supplementary Material), with sensitivities ranging from 0.12 to 0.19 and −0.19 to −0.10, respectively. At 100% obstruction, however, SpO_2_ was most sensitive to γ and the initial blood volume in the veins (V_V0_), with sensitivities of −6.11 and −4.95, respectively. Across all the obstruction levels, ETCO_2_ was most sensitive to the parameters representing the initial CO_2_ production rate in body tissue (M_dCO2_) and the fraction of dead space in the lungs (k_ds_), with sensitivities ranging from 0.83 to 1.02 and 0.36 to 0.55, respectively. For 0%–75% obstruction, both SBP and HR were most sensitive to V_V0_ and the parameter representing the initial unstressed blood volume in the veins (V_Vu0_), with sensitivities ranging from 0.60 to 1.54 and −1.39 to −0.57, respectively. However, at 100% obstruction, HR was most sensitive to the intercept term (HR_0_) and one of the gain terms (HR_2_) in the equation used to compute HR, with sensitivities of 0.55 and 0.44, respectively, while SBP remained most sensitive to V_V0_ and V_Vu0_, with sensitivities of 1.23 and −0.92, respectively. Thus, we identified eight model parameters (out of 163) that influenced four key vital signs. Supplementary Table S2 in the Supplementary Material shows the top two most sensitive parameters for the intermediate outputs MV and RR and their corresponding sensitivity values.

**TABLE 3:** List of the two most sensitive parameters for SpO_2_, ETCO_2_, SBP, and HR at five different airway obstruction levels.

Obstruction level (%)	Rank	SpO_2_		ETCO_2_		SBP		HR	
		Parameter	Sensitivity	Parameter	Sensitivity	Parameter	Sensitivity	Parameter	Sensitivity
0	1	γ	0.12	M_dCO2_	0.83	V_V0_	1.54	V_V0_	0.68
	2	P_50_	−0.10	k_ds_	0.55	V_Vu0_	−1.39	V_Vu0_	−0.63
25	1	γ	0.13	M_dCO2_	0.83	V_V0_	1.49	V_V0_	0.72
	2	P_50_	−0.10	k_ds_	0.55	V_Vu0_	−1.35	V_Vu0_	−0.67
50	1	γ	0.13	M_dCO2_	0.83	V_V0_	1.46	V_V0_	0.73
	2	P_50_	−0.11	k_ds_	0.53	V_Vu0_	−1.31	V_Vu0_	−0.68
75	1	P_50_	−0.19	M_dCO2_	0.83	V_V0_	1.29	V_V0_	0.60
	2	γ	0.19	k_ds_	0.45	V_Vu0_	−1.06	V_Vu0_	−0.57
100	1	γ	−6.11	M_dCO2_	1.02	V_V0_	1.23	HR_0_	0.55
	2	V_V0_	−4.95	k_ds_	0.36	V_Vu0_	−0.92	HR_2_	0.40

ETCO_2_, end-tidal carbon dioxide; HR, heart rate; HR_0_, intercept in the equation for HR computation; HR_2_, gain in the equation for HR computation; k_ds_, fraction of dead space in the lung; M_dCO2_, carbon dioxide production rate in the body tissue; P_50_, partial pressure of oxygen at which hemoglobin is 50% saturated; SBP, systolic blood pressure; SpO_2_, oxygen saturation; V_V0_, initial blood volume in the veins; V_Vu0_, initial unstressed blood volume in the veins; γ , Hill coefficient in the oxygen-hemoglobin dissociation sigmoidal function.

## 4 Discussion

Combat medics providing care in LSCO face numerous challenges, including high casualty rates, large casualty-to-provider ratios, limited medical resources and reach-back capability, and delayed evacuation. In such mass-casualty settings, AI technologies that can help optimize and accelerate medical decision-making will become increasingly essential to support timely and effective combat casualty care (Jin et al., 2024). The development of such technologies requires computational models that can accurately predict the human physiological response to relevant battlefield injuries and generate synthetic data to train the AI algorithms and evaluate their performance. Because airway obstruction is the second leading cause of potentially survivable death on the battlefield, we extended the CR model to incorporate its physiological effects (Eastridge et al., 2012; Eastridge et al., 2019). To this end, based on a model developed by Albanese et al. (2016), we added two new components to the CR model representing respiratory control and respiratory mechanics. The extended CR model can now simulate changes in nine key vital signs (SpO_2_, ETCO_2_, SBP, MAP, DBP, HR, MV, RR, and CO) in response to both hemorrhagic injury and airway obstruction, in addition to fluid resuscitation with six different fluid types and ketamine administration, thus expanding its capability to simulate a broader range of battlefield injuries and treatments.

To ensure that the extended CR model accurately represented airway obstruction, we assessed its performance at different stages of development. First, we validated only the newly added extensions (i.e., the respiratory control and mechanics components) by comparing MV predictions with experimental data involving changes in PaO_2_ and PaCO_2_ from *Studies 2* and *3*. On average, these components accurately predicted changes in MV, yielding an RMSE of 9.9 L/min with 100% of the predictions falling within 2 SEM of the experimental data (Table 2). However, their predictions of RR, which was only reported in *Study 3*, were less accurate, with an RMSE of 6.4 breaths/min and none of the predictions falling within 2 SEM of the three measured data points (Figure 4D). This discrepancy may be either due to the fact that we did not consider the RR output when we calibrated the parameters of the respiratory component or the limited number of experimental measurements. Yet, another possible explanation is the large variability in RR across different experimental studies, which limits the model’s ability to provide accurate predictions for individual cases. For example, *Study 6* also reported changes in RR in response to FiO_2_ and FiCO_2_ changes. When we simulated the protocol of *Study 6*, the CR-model predicted values for PaO_2_ (34–89 mmHg) and PaCO_2_ (30–41 mmHg) were actually similar to those used as inputs in *Study 3* (Table 1), yet *Study 3* reported much larger RR perturbations (10–22 breaths/min; Figure 4D) compared to *Study 6* (11–13 breaths/min; Figure 6D). In fact, in the case of *Study 6*, 100% of the CR-model predicted RR values fell within 2 SEM of the corresponding experimental data (Table 2). Overall, our implementation of the respiratory components reasonably captured changes in MV and RR in response to changes in PaO_2_ and PaCO_2_.

Next, we assessed the performance of the entire extended CR model, after integration of the respiratory components with the original model, by comparing its vital-sign predictions with experimental data from five different studies (*Studies 4–8*), which included three different scenarios: *1*) changes in FiO_2_ and FiCO_2_, *2*) airway obstruction, and *3*) hemorrhage followed by changes in ventilation. On average, across *Studies 4–8* (excluding the 100% airway obstruction in *Study 7*), the model demonstrated good agreement with the experimental data, achieving low RMSE values: 1.3 L/min for MV, 1.6 breaths/min for RR, 3.9% for SpO_2_, 2.4 mmHg for ETCO_2_, 7.8 mmHg for MAP, 5.1 mmHg for SBP, and 6.2 beats/min for HR (Table 2). In contrast, in *Study 7*, for the 100% airway-obstruction condition, the model yielded higher RMSE values for ETCO_2_, SBP, and HR. In our simulations, to be consistent with the experimental protocol, we assumed a 3-min period for this obstruction. However, because the vital signs (SpO_2_, ETCO_2_, SBP, and HR) changed very rapidly during this condition and it was challenging to determine the precise timing for the onset and release of the obstruction from the experimental data, even a small mismatch of a few seconds between the simulated and the experimental timings resulted in considerable differences in the vital signs. Apart from this condition in *Study 7*, as hypothesized, the extended CR model reasonably captured the vital-sign responses for a broad range of respiratory perturbations, with RMSEs for vital signs (MV, RR, SpO_2_, ETCO_2_, MAP, SBP, and HR) falling within 20% of their corresponding baseline values for an “average” human (6.6 L/min for MV, 12 breaths/min for RR, 98% for SpO_2_, 40 mmHg for ETCO_2_, 90 mmHg for MAP, 120 mmHg for SBP, and 70 beats/min for HR) (Hall, 2016).

We are aware of two other existing models that can simulate both hemorrhagic injury and airway obstruction and their associated treatments, HumMod (Hester et al., 2011) and Pulse Physiology Engine (Bray et al., 2019). However, these two models contain more than a thousand parameters and variables, making them too complex for seamless integration with AI algorithms. In addition, neither model has been quantitatively validated for airway-obstruction scenarios. In comparison, the CR model contains only 163 parameters and was quantitatively validated herein against experimental studies that represented relevant battlefield airway obstruction and its removal. Of note, we previously showed that, compared to HumMod, a simpler version of the CR model yielded similar or better performance in the prediction of vital-sign responses (SpO_2_, ETCO_2_, MAP, and HR) to hemorrhagic injury and airway perturbations (changes in MV and FiO_2_) (Jin et al., 2023).

Airway obstruction accounts for 8% of potentially survivable deaths on the battlefield and combined with hemorrhage contributes to 99% of the cases (Eastridge et al., 2012; Eastridge et al., 2019). In a study involving over 700 trauma casualties during combat operations in Afghanistan, 16.9% required some form of airway management in the prehospital setting (Blackburn et al., 2018). Airway obstruction directly impairs ventilation, leading to hypoxia and hypercapnia, which in turn negatively impact the function of the cardiovascular system. Knowledge of the location, duration, and severity of an airway obstruction and its associated interventions is important because it helps guide battlefield care, both in terms of the appropriate medical procedures to perform as well as medical logistics, e.g., availability of a cricothyrotomy kit or suction equipment. For example, the U.S. DoD Tactical Combat Casualty Care guidelines recommend frequent monitoring of SpO_2_ and ETCO_2_ to assess airway status and determine the appropriate airway management strategy (Deaton et al., 2021).

A key challenge in refining the guidelines for battlefield casualty care is the projection of future resource requirements based on the current physiological state of the casualties. AI algorithms trained on relevant battlefield data have demonstrated the capability to detect injuries, assess their severity, and recommend treatment strategies in real time (Jin et al., 2017; Jin et al., 2018; Fernandes et al., 2020; Dolan et al., 2021; Maurer et al., 2021; Peng et al., 2023; Stallings et al., 2023; Jin et al., 2024). However, the development of such AI algorithms requires large amounts of clinical data, preferably those capturing relevant battlefield injuries, such as simultaneous hemorrhagic and airway-obstruction injuries. Without clinical data, we must rely on synthetic data. In fact, using an earlier version of the CR model, we previously generated a synthetic hemorrhagic-injury trauma dataset and subsequently used it to develop an AI algorithm to recommend optimal fluid allocation strategies for a variety of simulated mass-casualty scenarios (Jin et al., 2024). Compared to the current U.S. DoD standard of care guidelines for blood transfusion [the Vampire Program (Voller et al., 2021)], computer simulations showed that this AI algorithm could restore 46% more casualties to healthy vital-sign levels and could increase fluid-utilization efficiency by nearly 120%. With the recent extensions to the CR model, including the effects of different fluid types (Kurian et al., 2025), ketamine administration, and the current capability to simulate airway obstruction, we have considerably increased the scope of injuries and associated treatment options that the extended CR model can simulate. In the future, one potential use of the extended model would be to generate a much larger and diverse set of synthetic data of trauma casualties and use it to train AI algorithms to optimize the management of multiple injury types. Another potential use could involve its integration into a model-based decision-support system (Glachs et al., 2021), where the CR model enhanced by a parameter-estimation extended Kalman filter algorithm (Laxminarayan et al., 2023) would assess the efficacy of different treatment options in real time (e.g., nasal high-flow ventilation, mask ventilation, tracheal intubation, tracheotomy, laryngeal mask ventilation, and mechanical ventilation) to provide optimal personalized recommendations.

Although by and large the extended CR model captured the variations in vital signs resulting from airway obstruction, it does have several limitations due to simplifying assumptions we had to make during model development. First, we validated the CR model using experimental data from pig studies. We attempted to overcome this limitation by normalizing the experimental inputs to an average human. However, there could be potential species-specific differences in respiratory mechanics between pigs and humans that we cannot overcome by data normalization. For example, during hypercapnic acidosis, the diaphragm muscle’s ability to contract is preserved in pigs but impaired in humans, which could lead to a decrease in tidal volume and MV in humans (Morales-Quinteros et al., 2019). Therefore, by not considering species-specific differences, our predictions of MV, for example, could be less accurate. Second, without considering a specific cause, we modeled airway obstruction as an increase in airway resistance caused by a decrease in airway diameter, akin to a blockage, at three representative locations along the upper and lower airways. In addition, we did not consider the individual compliances of the larynx, trachea, and bronchi in the model predictions. However, depending on its cause, airway obstruction can have distinct clinical manifestations, such as airway edema or external hematoma, which can lead to dynamic changes in airway compliance (Martin-Lefevre et al., 2001; Palmer and Clegg, 2023). Thus, our simplified representation of airway obstruction could lead to over- or under-estimation of changes in vital signs.

Third, we used a simplified model of respiratory rhythm generation, as described in Albanese et al., which does not account for the physiological variability in respiratory rhythm or the changes in airway tone that occur during stress and trauma (Butera et al., 1999a; Butera et al., 1999b; Smith et al., 2000; Elstad et al., 2018; Baertsch et al., 2021). We had to make this simplifying assumption because our model currently does not possess the granularity (i.e., the description of the sympathetic and parasympathetic neurons that signal the smooth muscle cells to contract or dilate) to incorporate these changes in airway tone. In the future, the representation of the effects of such dynamic changes in airway tone and variability in the respiratory rhythm will allow us to more accurately capture the clinical responses of airway injuries. Fourth, the current CR model does not account for environmental factors, such as ambient temperature and relative humidity. For example, inhalation of cold air can lead to airway narrowing, which leads to increased airway resistance in real life (Fontanari et al., 1996). We excluded environmental factors because we currently do not have enough data to model these factors. When such data become available, we will incorporate them in the model to increase its accuracy. Lastly, the model currently does not incorporate certain battlefield injuries that affect the cardiovascular and respiratory systems, such as smoke inhalation or burns. These injuries, while important, are not among the leading causes of potentially survivable deaths on the battlefield, so we did not prioritize them in the current model.

In summary, we extended and validated our previously developed CR model to predict the temporal changes in vital signs caused by airway obstruction. This new capability broadens the scope of the injury and treatment scenarios that the CR model can simulate and allows us to generate synthetic trauma casualty datasets that include hemorrhagic injury, airway obstruction, fluid resuscitation with saline, whole blood, and blood products, and the administration of pain medication. Such datasets are essential for the development of AI algorithms to help combat medics triage, diagnose, and treat combat casualties near the point of injury.

## Data Availability

The original contributions presented in the study are included in the article/Supplementary Material, further inquiries can be directed to the corresponding author.

## References

[B1] Albanese A. Cheng L. Ursino M. Chbat N. W. (2016). An integrated mathematical model of the human cardiopulmonary system: model development. Am. J. Physiol. Heart Circ. Physiol. 310 (7), H899–H921. 10.1152/ajpheart.00230.2014 26683899

[B2] April M. D. Schauer S. G. Long B. Hood L. De Lorenzo R. A. (2023). Airway management during large-scale combat operations: a narrative review of capability requirements. Med. J. (Ft Sam Houst Tex) (Per 23-1/2/3), 18–27. 36607294

[B3] Baertsch N. A. Bush N. E. Burgraff N. J. Ramirez J. M. (2021). Dual mechanisms of opioid-induced respiratory depression in the inspiratory rhythm-generating network. Elife 10, e67523. 10.7554/eLife.67523 34402425 PMC8390004

[B4] Benito S. Lemaire F. Mankikian B. Harf A. (1985). Total respiratory compliance as a function of lung volume in patients with mechanical ventilation. Intensive Care Med. 11 (2), 76–79. 10.1007/BF00254778 3989101

[B5] Bighamian R. Kinsky M. Kramer G. Hahn J. O. (2017). In-human subject-specific evaluation of a control-theoretic plasma volume regulation model. Comput. Biol. Med. 91, 96–102. 10.1016/j.compbiomed.2017.10.006 29049911

[B6] Blackburn M. B. April M. D. Brown D. J. Delorenzo R. A. Ryan K. L. Blackburn A. N. (2018). Prehospital airway procedures performed in trauma patients by ground forces in Afghanistan. J. Trauma Acute Care Surg. 85 (1S Suppl. 2), S154-S160–S160. 10.1097/TA.0000000000001866 29521802

[B7] Blackburn M. B. Nawn C. D. Ryan K. L. (2019). Testing of novel spectral device sensor in swine model of airway obstruction. Physiol. Rep. 7 (19), e14246. 10.14814/phy2.14246 31587488 PMC6778596

[B8] Blackburn M. B. Hudson I. L. Rodriguez C. Wienandt N. Ryan K. L. (2021). Acute overventilation does not cause lung damage in moderately hemorrhaged swine. J. Appl. Physiol. 130 (5), 1337–1344. 10.1152/japplphysiol.01048.2020 33734830

[B9] Bray A. Webb J. B. Enquobahrie A. Vicory J. Heneghan J. Hubal R. (2019). Pulse physiology engine: an open-source software platform for computational modeling of human medical simulation. SN Comp. Clin. Med. 1, 362–377. 10.1007/s42399-019-00053-w

[B10] Breeze J. Bryant D. (2009). Current concepts in the epidemiology and management of battlefield head, face and neck trauma. BMJ Mil. Health 155, 274–278. 10.1136/jramc-155-04-07 20397602

[B11] Butera R. J. Jr. Rinzel J. Smith J. C. (1999a). Models of respiratory rhythm generation in the pre-botzinger complex. I. Bursting pacemaker neurons. J. Neurophysiol. 82 (1), 382–397. 10.1152/jn.1999.82.1.382 10400966

[B12] Butera R. J. Jr. Rinzel J. Smith J. C. (1999b). Models of respiratory rhythm generation in the pre-botzinger complex. II. Populations of coupled pacemaker neurons. J. Neurophysiol. 82 (1), 398–415. 10.1152/jn.1999.82.1.398 10400967

[B13] Cheng L. Albanese A. Ursino M. Chbat N. W. (2016). An integrated mathematical model of the human cardiopulmonary system: model validation under hypercapnia and hypoxia. Am. J. Physiol. Heart Circ. Physiol. 310 (7), H922–H937. 10.1152/ajpheart.00923.2014 26747507

[B14] Chiari L. Avanzolini G. Ursino M. (1997). A comprehensive simulator of the human respiratory system: validation with experimental and simulated data. Ann. Biomed. Eng. 25 (6), 985–999. 10.1007/BF02648124 9395044

[B15] Deaton T. G. Auten J. D. Betzold R. Butler F. K. Jr. Byrne T. Cap A. P. (2021). Fluid resuscitation in tactical combat casualty care; TCCC guidelines change 21-01. 4 November 2021. J. Spec. Oper. Med. 21 (4), 126–137. 10.55460/JYLU-4OZ8 34969143

[B16] Dolan C. P. Valerio M. S. Lee Childers W. Goldman S. M. Dearth C. L. (2021). Prolonged field care for traumatic extremity injuries: defining a role for biologically focused technologies. NPJ Regen. Med. 6 (1), 6. 10.1038/s41536-020-00117-9 33542235 PMC7862384

[B17] Dorrington K. L. Clar C. Young J. D. Jonas M. Tansley J. G. Robbins P. A. (1997). Time course of the human pulmonary vascular response to 8 hours of isocapnic hypoxia. Am. J. Physiol. Heart Circ. Physiol. 273 (3), H1126–H1134. 10.1152/ajpheart.1997.273.3.H1126 9321798

[B18] Eastridge B. J. Mabry R. L. Seguin P. Cantrell J. Tops T. Uribe P. (2012). Death on the battlefield (2001-2011): implications for the future of combat casualty care. J. Trauma Acute Care Surg. 73 (6 Suppl. 5), S431–S437. 10.1097/TA.0b013e3182755dcc 23192066

[B19] Eastridge B. J. Holcomb J. B. Shackelford S. (2019). Outcomes of traumatic hemorrhagic shock and the epidemiology of preventable death from injury. Transfusion 59 (S2), 1423–1428. 10.1111/trf.15161 30980749

[B20] Ellwein L. M. Pope S. R. Xie A. Batzel J. J. Kelley C. T. Olufsen M. S. (2013). Patient-specific modeling of cardiovascular and respiratory dynamics during hypercapnia. Math. Biosci. 241 (1), 56–74. 10.1016/j.mbs.2012.09.003 23046704 PMC4183199

[B21] Elstad M. O'Callaghan E. L. Smith A. J. Ben-Tal A. Ramchandra R. (2018). Cardiorespiratory interactions in humans and animals: rhythms for life. Am. J. Physiol. Heart Circ. Physiol. 315 (1), H6–H17. 10.1152/ajpheart.00701.2017 29522373

[B22] Epstein A. Lim R. Johannigman J. Fox C. J. Inaba K. Vercruysse G. A. (2023). Putting medical boots on the ground: lessons from the war in Ukraine and applications for future conflict with near-peer adversaries. J. Am. Coll. Surg. 237 (2), 364–373. 10.1097/XCS.0000000000000707 37459197 PMC10344429

[B23] Fernandes M. Vieira S. M. Leite F. Palos C. Finkelstein S. Sousa J. M. C. (2020). Clinical decision support systems for triage in the emergency department using intelligent systems: a review. Artif. Intell. Med. 102, 101762. 10.1016/j.artmed.2019.101762 31980099

[B24] Fink M. Batzel J. J. Kappel F. (2004). An optimal control approach to modeling the cardiovascular-respiratory system: an application to orthostatic stress. Cardiovasc. Eng. 4 (1), 27–38. 10.1023/B:CARE.0000025120.30148.7a

[B25] Fontanari P. Burnet H. Zattara-Hartmann M. C. Jammes Y. (1996). Changes in airway resistance induced by nasal inhalation of cold dry, dry, or moist air in normal individuals. J. Appl. Physiol. 81 (4), 1739–1743. 10.1152/jappl.1996.81.4.1739 8904594

[B26] Gerling K. A. Kersey A. J. Lauria A. L. Mares J. A. Hutzler J. D. White P. W. (2023). Evaluation of novel hemostatic agents in a coagulopathic swine model of junctional hemorrhage. J. Trauma Acute Care Surg. 95 (2S Suppl. 1), S144–S151. 10.1097/TA.0000000000004071 37259206 PMC10389358

[B27] Gibbons A. J. Breeze A. (2011). The face of war: the initial management of modern battlefield ballistic facial injuries. J. Mil. Veterans Health 19 (2), 15–18.

[B28] Glachs D. Namli T. Strohmeier F. Rodriguez Suarez G. Sluis M. Delgado-Lista J. (2021). A predictive model-based decision support system for diabetes patient empowerment. Stud. Health Technol. Inf. 281, 963–968. 10.3233/SHTI210321 34042816

[B29] Gourine A. V. (2005). On the peripheral and central chemoreception and control of breathing: an emerging role of ATP. J. Physiol. 568, 715–724. 10.1113/jphysiol.2005.095968 16141266 PMC1464180

[B30] Griffiths D. F. Higham D. J. (2010). Numerical methods for ordinary differential equations: initial value problems. New York, NY: Springer.

[B31] Hall J. E. (2016). Guyton and Hall Textbook of Medical Physiology. Amsterdam, Netherlands: Elsevier Health Sciences.

[B32] Hampson N. B. Camporesi E. Stolp B. Moon R. Shook J. Griebel J. (1990). Cerebral oxygen availability by NIR spectroscopy during transient hypoxia in humans. J. Appl. Physiol. 69 (3), 907–913. 10.1152/jappl.1990.69.3.907 2174031

[B33] Helton J. C. Davis F. J. (2003). Latin hypercube sampling and the propagation of uncertainty in analyses of complex systems. Reliab. Eng. Syst. Saf. 81 (1), 23–69. 10.1016/S0951-8320(03)00058-9

[B34] Hessert M. J. Bennett B. L. (2013). Optimizing emergent surgical cricothyrotomy for use in austere environments. Wilderness Environ. Med. 24 (1), 53–66. 10.1016/j.wem.2012.07.003 23062323

[B35] Hester R. L. Brown A. J. Husband L. Iliescu R. Pruett D. Summers R. (2011). HumMod: a modeling environment for the simulation of integrative human physiology. Front. Physiol. 2, 12. 10.3389/fphys.2011.00012 21647209 PMC3082131

[B36] Jin X. Kim C. S. Dumont G. A. Ansermino J. M. Hahn J. O. (2017). A semi‐adaptive control approach to closed‐loop medication infusion. Int. J. Adapt Control Signal Process 31 (2), 240–254. 10.1002/acs.2696

[B37] Jin X. Bighamian R. Hahn J. O. (2018). Development and *in silico* evaluation of a model-based closed-loop fluid resuscitation control algorithm. IEEE Trans. Biomed. Eng. 66 (7), 1905–1914. 10.1109/TBME.2018.2880927 30452347

[B38] Jin X. Laxminarayan S. Nagaraja S. Wallqvist A. Reifman J. (2023). Development and validation of a mathematical model to simulate human cardiovascular and respiratory responses to battlefield trauma. Int. J. Numer. Method Biomed. Eng. 39 (1), e3662. 10.1002/cnm.3662 36385572

[B39] Jin X. Frock A. Nagaraja S. Wallqvist A. Reifman J. (2024). AI algorithm for personalized resource allocation and treatment of hemorrhage casualties. Front. Physiol. 15, 1327948. 10.3389/fphys.2024.1327948 38332989 PMC10851938

[B40] Karavaev A. S. Ishbulatov Y. M. Ponomarenko V. I. Prokhorov M. D. Gridnev V. I. Bezruchko B. P. (2016). Model of human cardiovascular system with a loop of autonomic regulation of the mean arterial pressure. J. Am. Soc. Hypertens. 10 (3), 235–243. 10.1016/j.jash.2015.12.014 26847603

[B41] Kim S. J. Koh K. Boyd S. Gorinevsky D. (2009). l_1_ trend filtering. SIAM Rev. 51 (2), 339–360. 10.1137/070690274

[B42] Krausz A. A. Krausz M. M. Picetti E. (2015). Maxillofacial and neck trauma: a damage control approach. World J. Emerg. Surg. 10 (1), 31. 10.1186/s13017-015-0022-9 26157475 PMC4495937

[B43] Kronenberg R. Hamilton F. N. Gabel R. Hickey R. Read D. J. Severinghaus J. (1972). Comparison of three methods for quantitating respiratory response to hypoxia in man. Respir. Physiol. 16 (1), 109–125. 10.1016/0034-5687(72)90092-8 5073532

[B44] Kurian V. Jin X. Nagaraja S. Wallqvist A. Reifman J. (2025). A model to simulate human cardio-respiratory responses to different fluid resuscitation treatments after hemorrhagic injury. Front. Physiol. 16, 1613874. 10.3389/fphys.2025.1613874 40666119 PMC12259663

[B45] Laxminarayan S. Hornby S. Belval L. N. Giersch G. E. W. Morrissey M. C. Casa D. J. (2023). Prospective validation of *2B-Cool*: integrating wearables and individualized predictive analytics to reduce heat injuries. Med. Sci. Sports Exerc. 55 (4), 751–764. 10.1249/MSS.0000000000003093 36730025

[B46] Longobardo G. S. Evangelisti C. J. Cherniack N. S. (2008). Analysis of the interplay between neurochemical control of respiration and upper airway mechanics producing upper airway obstruction during sleep in humans. Exp. Physiol. 93 (2), 271–287. 10.1113/expphysiol.2007.039917 17933858

[B47] Lurin I. A. Khomenko I. P. Kashtalyan M. Mcknight G. Nehoduyko V. V. Tertyshnyi S. V. (2025). A novel approach used for reconstruction of facial blast wound injury-a case report from the russo-ukrainian war. J. Surg. Case Rep. 2025 (3), rjae709. 10.1093/jscr/rjae709 40051809 PMC11881685

[B48] Lutfi M. F. (2017). The physiological basis and clinical significance of lung volume measurements. Multidiscip. Respir. Med. 12 (1), 3. 10.1186/s40248-017-0084-5 28194273 PMC5299792

[B49] Mabry R. L. Edens J. W. Pearse L. Kelly J. F. Harke H. (2010). Fatal airway injuries during operation enduring Freedom and operation Iraqi freedom. Prehosp. Emerg. Care 14 (2), 272–277. 10.3109/10903120903537205 20199236

[B50] Martin-Gill C. Wheeler B. J. Guyette F. X. Wheeler S. E. (2024). Correlation between EtCO_2_ and PCO_2_ in patients undergoing critical care transport. Prehosp. Emerg. Care 29, 953–961. 10.1080/10903127.2024.2430394 39546437

[B51] Martin-Lefevre L. Ricard J. D. Roupie E. Dreyfuss D. Saumon G. (2001). Significance of the changes in the respiratory system pressure-volume curve during acute lung injury in rats. Am. J. Respir. Crit. Care Med. 164 (4), 627–632. 10.1164/ajrccm.164.4.2008018 11520727

[B52] Maurer L. R. Bertsimas D. Bouardi H. T. El Hechi M. El Moheb M. Giannoutsou K. (2021). Trauma outcome predictor: an artificial intelligence interactive smartphone tool to predict outcomes in trauma patients. J. Trauma Acute Care Surg. 91 (1), 93–99. 10.1097/TA.0000000000003158 33755641

[B53] Mecklenburgh J. S. Mapleson W. W. (1998). Ventilatory assistance and respiratory muscle activity. 2: simulation with an adaptive active (“aa” or “a-squared”) model lung. Br. J. Anaesth. 80 (4), 434–439. 10.1093/bja/80.4.434 9640145

[B54] Mitrophanov A. Y. Churchward G. Borodovsky M. (2007). Control of *Streptococcus pyogenes* virulence: modeling of the CovR/S signal transduction system. J. Theor. Biol. 246 (1), 113–128. 10.1016/j.jtbi.2006.11.009 17240398 PMC2688695

[B55] Morales-Quinteros L. Camprubi-Rimblas M. Bringue J. Bos L. D. Schultz M. J. Artigas A. (2019). The role of hypercapnia in acute respiratory failure. Intensive Care Med. Exp. 7 (Suppl. 1), 39. 10.1186/s40635-019-0239-0 31346806 PMC6658637

[B56] Palmer B. F. Clegg D. J. (2023). Respiratory acidosis and respiratory alkalosis: core curriculum 2023. Am. J. Kidney Dis. 82 (3), 347–359. 10.1053/j.ajkd.2023.02.004 37341662

[B57] Peng H. T. Siddiqui M. M. Rhind S. G. Zhang J. Da Luz L. T. Beckett A. (2023). Artificial intelligence and machine learning for hemorrhagic trauma care. Mil. Med. Res. 10 (1), 6. 10.1186/s40779-023-00444-0 36793066 PMC9933281

[B58] Prysiazhniuk O. Palyvoda R. Chepurnyi Y. Pavlychuk T. Chernogorskyi D. Fedirko I. (2025). War-related maxillofacial injuries in Ukraine: a retrospective multicenter study. Arch. Craniofac. Surg. 26 (2), 51–58. 10.7181/acfs.2024.0074 40335049 PMC12061781

[B59] Reynolds W. J. Milhorn H. T. Jr. (1973). Transient ventilatory response to hypoxia with and without controlled alveolar PCO_2_ . J. Appl. Physiol. 35 (2), 187–196. 10.1152/jappl.1973.35.2.187 4723026

[B60] Reynolds W. J. Milhorn H. T. Jr. Holloman G. H. Jr. (1972). Transient ventilatory response to graded hypercapnia in man. J. Appl. Physiol. 33 (1), 47–54. 10.1152/jappl.1972.33.1.47 5037410

[B61] Ross J. D. Burns C. J. Sagini E. M. Zarzabal L. A. Morrison J. J. (2014). A laparoscopic swine model of noncompressible torso hemorrhage. J. Trauma Acute Care Surg. 77 (3 Suppl. 2), S77–S82. 10.1097/TA.0000000000000385 25159366

[B62] Smith J. C. Butera R. J. Jr. Koshiya N. Del Negro C. Wilson C. G. Johnson S. M. (2000). Respiratory rhythm generation in neonatal and adult mammals: the hybrid pacemaker-network model. Respir. Physiol. 122 (2–3), 131–147. 10.1016/s0034-5687(00)00155-9 10967340

[B63] Sondeen J. L. Prince M. D. Kheirabadi B. S. Wade C. E. Polykratis I. A. De Guzman R. (2011). Initial resuscitation with plasma and other blood components reduced bleeding compared to hetastarch in anesthetized swine with uncontrolled splenic hemorrhage. Transfusion 51 (4), 779–792. 10.1111/j.1537-2995.2010.02928.x 21091492

[B64] Stallings J. D. Laxminarayan S. Yu C. Kapela A. Frock A. Cap A. P. (2023). APPRAISE-HRI: an artificial intelligence algorithm for triage of hemorrhage casualties. Shock 60 (2), 199–205. 10.1097/SHK.0000000000002166 37335312 PMC10476583

[B65] Szpinda M. Siedlaczek W. Szpinda A. Woźniak A. L. Mila-Kierzenkowska C. Wiśniewski M. (2014). Volumetric growth of the lungs in human fetuses: an anatomical, hydrostatic and statistical study. Surg. Radiol. Anat. 36 (8), 813–820. 10.1007/s00276-014-1269-7 24535661 PMC4171590

[B66] Trenhago P. R. Fernandes L. G. Muller L. O. Blanco P. J. Feijoo R. A. (2016). An integrated mathematical model of the cardiovascular and respiratory systems. Int. J. Numer. Method Biomed. Eng. 32 (1), e02736. 10.1002/cnm.2736 26198626

[B67] Tsur N. Dudkiewicz D. Talmy T. Radomislensky I. Givon A. Katorza E. (2025). Battlefield neck injuries: contemporary insights from the Israeli national trauma registry. J. Am. Coll. Emerg. Physicians Open 6 (4), 100211. 10.1016/j.acepjo.2025.100211 40641888 PMC12242401

[B68] United States Army Medical Center of Excellence Lessons Learned Branch (2025). Ukraine medical lessons learned report. Available online at: https://www.lineofdeparture.army.mil/Journals/Pulse-of-Army-Medicine/Archive/June-2025/UKRAINE-MEDICAL-LESSONS/(accessed on October 14, 2025).

[B69] Voller J. Tobin J. M. Cap A. P. Cunningham C. W. Denoyer M. Drew B. (2021). Joint Trauma System clinical practice guideline (JTS CPG): prehospital blood transfusion. 30 October 2020. J. Spec. Oper. Med. 21 (4), 11–21. 10.55460/P685-L7R7 34969121

[B70] Weil J. V. Byrne-Quinn E. Sodal I. E. Friesen W. O. Underhill B. Filley G. F. (1970). Hypoxic ventilatory drive in normal man. J. Clin. Invest. 49, 1061–1072. 10.1172/JCI106322 5422012 PMC322574

[B71] Ziebart A. Garcia-Bardon A. Kamuf J. Thomas R. Liu T. Schad A. (2015). Pulmonary effects of expiratory-assisted small-lumen ventilation during upper airway obstruction in pigs. Anaesthesia 70 (10), 1171–1179. 10.1111/anae.13154 26179167

