# Supplementary material for: A model to simulate human cardio-respiratory responses to airway obstruction
Source: Front Physiol. 2025 Nov 28;16:1699315. doi: 10.3389/fphys.2025.1699315 (PMC12698400; doi:10.3389/fphys.2025.1699315)
Supplement: Supplementary file 1 [file DataSheet1.docx]

***Supplementary Material***

A model to simulate human cardio-respiratory responses to airway obstruction

Xin Jin^1,2^, Varghese Kurian^1,2^, Kathy L. Ryan^3^, Anders Wallqvist^1^, Jaques Reifman^1*^, and Sridevi Nagaraja^1,2^

^1^Department of Defense Biotechnology High Performance Computing Software Applications Institute, Defense Health Agency Research & Development, Medical Research and Development Command, Fort Detrick, MD, USA

^2^The Henry M. Jackson Foundation for the Advancement of Military Medicine, Inc., Bethesda, MD, USA

^3^United States Army Institute of Surgical Research, Fort Sam Houston, San Antonio, TX, USA

Table S1. Complete list of parameters used in the cardio-respiratory model.

1. Cardiovascular model

| **P#** | **Parameter name** | **Description** | **Value** | **Units** | **Source** |
| --- | --- | --- | --- | --- | --- |
| 1 | V_V0_ | Initial blood volume in the veins | 3.26 | L | Guyton (1980) |
| 2 | V_Vu0_ | Initial unstressed blood volume in the veins | 2.95 | L |  |
| 3 | C_v_ | Venous compliance | 8.25$\text{×}$10^−2^ | L/mmHg |  |
| 4 | R_v0_ | Initial venous resistance | 0.74 | mmHg$\text{·}$min/L |  |
| 5 | V_ra0_ | Initial blood volume in the right atrium | 0.10 | L |  |
| 6 | V_rau_ | Unstressed blood volume in the right atrium | 0.10 | L |  |
| 7 | C_ra_ | Right atrial compliance | 5.00$\text{×}$10^−2^ | L/mmHg |  |
| 8 | V_a0_ | Initial blood volume in the arteries | 0.85 | L |  |
| 9 | V_au_ | Unstressed blood volume in the arteries | 0.50 | L |  |
| 10 | C_a_ | Arterial compliance | 3.60$\text{×}$10^−3^ | L/mmHg |  |
| 11 | R_a0_ | Initial arterial resistance | 19.34 | mmHg$\text{·}$min/L |  |
| 12 | HS_0_ | Initial heart strength | 1.00 | - |  |
| 13 | k_SV_ | Gain of the sympathetic effects on V_V0_ | 0.25 | L |  |
| 14 | k_SHS_ | Gain of the sympathetic effects on heart strength | 1.00 | - |  |
| 15 | k_SR_ | Gain of the sympathetic effects on R_a0_ | 1.00 | - |  |
| 16 | k_Sk_ | Gain of the sympathetic effects on kidney function | 1.00 | - |  |
| 17 | k_AV_ | Gain of the angiotensin effects on V_V0_ | 0.20 | L |  |
| 18 | k_AR_ | Gain of the angiotensin effects on R_a0_ | 1.00 | - |  |
| 19 | HR_0_ | Intercept in the equation for heart rate computation | 32.00 | beats/min |  |
| 20 | HR_1_ | Gains in the equation for heart rate computation | 2.00 | beats/  (min$\text{·}$mmHg) |  |
| 21 | HR_2_ | Gains in the equation for heart rate computation | 40.00 | beats/min |  |
| 22 | $\text{k}\text{cp}$ | Factor used in the calculation of capillary pressure | 2.80 | mmHg$\text{·}$min/L | Kurian et al. (2025a) |
| 23 | $\text{k}_{\text{ab}}$ | Factor used to scale the pressure acting on the baroreceptor | 0.65 | - |  |
| 24 | $\text{k}_{\text{sym}}$ | Sensitivity of the sympathetic stimulation | 1.50 | - |  |
| 25 | $\text{k}_{\text{ang}}$ | Factor used to scale the pressure acting on the angiotensin system | 0.45 | - |  |
| 26 | $\text{k}_{\text{angs}}$ | Sensitivity of the angiotensin system | 1.20 | - |  |
| 27 | $\text{A}_{\text{min}}$ | Lower limit of autoregulation | 2.19 | L/min |  |
| 28 | $\text{σ}^{\text{salt}}$ | Reflection coefficient of salt | 1.30$\text{×}$10^−2^ | - |  |
| 29 | $\text{σ}^{\text{alb}}$ | Reflection coefficient of albumin | 0.95 | - | Mazzoni et al. (1988) |
| 30 | $\text{L}_{\text{Pl-EC}}$ | Hydraulic conductivity of endothelial cells | 8.49$\text{×}$10^−10^ | L/(min $\text{·}$mmHg$\text{·}$cm^2^) |  |
| 31 | $\text{L}_{\text{Pl-ISS}}$ | Hydraulic conductivity of pores | 1.35$\text{×}$10^−6^ | L/(min $\text{·}$mmHg$\text{·}$cm^2^) | Kurian et al. (2025a) |
| 32 | $\text{L}_{\text{Pl-RBC}}$ | Hydraulic conductivity of red blood cells | 7.20$\text{×}$10^−10^ | L/(min $\text{·}$mmHg$\text{·}$cm^2^) | Mazzoni et al. (1988) |
| 33 | $\text{L}_{\text{ISS-}\text{Tcell}}$ | Hydraulic conductivity of tissue cells | 1.30$\text{×}$10^−9^ | L/(min $\text{·}$mmHg$\text{·}$cm^2^) |  |
| 34 | $\text{S}_{\text{Pl-EC}}$ | Surface area of the endothelial cell layer | 2.94$\text{×}$10^6^ | cm^2^ |  |
| 35 | $\text{S}_{\text{Pl-ISS}}$ | Surface area of pores | 2.94$\text{×}$10^4^ | cm^2^ |  |
| 36 | $\text{S}_{\text{Pl-RBC}}$ | Surface area of red blood cells | 1.35$\text{×}$10^2^ | μm^2^/cell |  |
| 37 | $\text{S}_{\text{ISS-}\text{Tcell}}$ | Surface area of tissue cells | 8.82$\text{×}$10^6^ | cm^2^ |  |
| 38 | $\text{J}_{\text{L0}}^{\text{w}}$ | Lymphatic flow at nominal conditions | 1.60$\text{×}$10^−3^ | L/min |  |
| 39 | $\text{P}\text{S}_{\text{Pl-ISS}}^{\text{salt}}$ | Permeability surface area product of salt | 2.50 | L/min |  |
| 40 | $\text{P}\text{S}_{\text{Pl-ISS}}^{\text{alb}}$ | Permeability surface area product of albumin | 2.50$\text{×}$10^−3^ | L/min | Kurian et al. (2025a) |
| 41 | $\text{V}_{\text{refill}}^{\text{max}}$ | Upper bound on transcapillary refill | 0.94 | L |  |
| 42 | k_bp_ | Coefficient for systolic and diastolic pressure | 3.85$\text{×}$10^2^ | mmHg$\text{·}$beats/L |  |
| 43 | $\text{τ}\text{sym}$ | Time constant of stimulated sympathetic activity | 0.26 | min |  |
| 44 | $\text{τ}\text{ang}$ | Time constant of angiotensin response | 6.44 | min |  |

1. Respiratory model

| **P#** | **Parameter name** | **Description** | **Value** | **Units** | **Source** |
| --- | --- | --- | --- | --- | --- |
| 45 | V_d(i)_, i = 1,2,…,5 | Volume of i^th^ dead space compartment | 0.03 | L | Cheng et al. (2010) |
| 46 | V_A_ | Volume of the lungs | 3.28 | L | Ursino et al. (2001) |
| 47 | s | Shunt fraction | 3.80$\text{×}$10^−2^ | - | Cheng et al. (2010) |
| 48 | V_b_ | Volume of the brain tissue | 1.26 | L |  |
| 49 | V_d_ | Volume of the body tissue | 42.00 | L |  |
| 50 | M_bO2_ | Initial O_2_ consumption rate in the brain tissue | 3.80$\text{×}$10^−2^ | L/min |  |
| 51 | M_dO2_ | Initial O_2_ consumption rate in the body tissue | 0.26 | L/min |  |
| 52 | M_bCO2_ | Initial CO_2_ production rate in the brain tissue | 3.84$\text{×}$10^−2^ | L/min |  |
| 53 | M_dCO2_ | Initial CO_2_ production rate in the body tissue | 0.17 | L/min |  |
| 54 | $\text{α}_{\text{1}}$ | Parameters in the equation for O_2_ and CO_2_ pressures and concentrations | 0.04 | 1/mmHg |  |
| 55 | $\text{β}_{\text{1}}$ |  | 8.00$\text{×}$10^−2^ | 1/mmHg |  |
| 56 | k_1_ |  | 14.00 | mmHg |  |
| 57 | $\text{α}_{\text{2}}$ |  | 5.60$\text{×}$10^−2^ | 1/mmHg |  |
| 58 | $\text{β}_{\text{2}}$ |  | 3.20$\text{×}$10^−2^ | 1/mmHg |  |
| 59 | k_2_ |  | 1.94$\text{×}$10^2^ | mmHg |  |
| 60 | k_ds_ | Fraction of dead space in the lungs | 0.35 | - | Ursino et al. (2001) |
| 61 | $\text{λ}$ | Coefficient that converts blood concentration into alveolar partial pressure | 8.63$\text{×}$10^2^ | mmHg |  |
| 62 | $\text{α}_{\text{O2}}$ | O_2_ solubility in the brain and body tissues | 3.17$\text{×}$10^−5^ | 1/mmHg |  |
| 63 | $\text{α}_{\text{CO2}}$ | CO_2_ solubility in the brain and body tissues | 6.67$\text{×}$10^−4^ | 1/mmHg |  |
| 64 | $\text{a}_{\text{1}}$ | Coefficients in the equation for O_2_ and CO_2_ pressures and concentrations | 0.38 | - |  |
| 65 | $\text{a}_{\text{2}}$ |  | 1.80 | - |  |
| 66 | $\text{c}_{\text{1}}$ |  | 9.00 | mmol/L |  |
| 67 | $\text{c}_{\text{2}}$ |  | 86.11 | mmol/L |  |
| 68 | P_50_ | Partial pressure of O_2_ at which hemoglobin is 50% saturated | 26.40 | mmHg | Cheng et al. (2010) |
| 69 | $\text{γ}$ | Hill coefficient in the O_2_-hemoglobin dissociation sigmoidal function | 2.65 | - |  |

1. Control due to chemoreceptors

| **P#** | **Parameter name** | **Description** | **Value** | **Units** | **Source** |
| --- | --- | --- | --- | --- | --- |
| 70 | f_acmin_ | Lower saturation level of frequency discharge in the afferent chemoreceptor | 50.00 | spikes/min | Cheng et al. (2010) |
| 71 | f_acmax_ | Upper saturation level of frequency discharge in the afferent chemoreceptor | 7.38$\text{×}$10^2^ | spikes/min |  |
| 72 | $\tilde{\text{P}}$_O2ac_ | Arterial O_2_ pressure at the central point of the afferent chemoreceptor | 45.00 | mmHg |  |
| 73 | k_ac_ | Parameters in the afferent chemoreceptor response | 29.28 | mmHg |  |
| 74 | f_0_ |  | 1.40 | - |  |
| 75 | K_H_ |  | 3.00 | - |  |
| 76 | G_ap_ | Gain of the pulmonary receptor response | 1.40$\text{×}$10^3^ | spikes/min/L |  |
| 77 | x_sh_ | Parameters in the offset terms of the sympathetic neural activation equation | 3.18$\text{×}$10^3^ | 1/min |  |
| 78 | $\tilde{\text{P}}$_O2sh_ |  | 45.00 | mmHg |  |
| 79 | k_sh_ |  | 6.00 | mmHg |  |
| 80 | g_sh_ |  | 60.00 | 1/(mmHg$\text{·}$min) |  |
| 81 | x_sr_, x_sv_ |  | 3.60$\text{×}$10^2^ | 1/min |  |
| 82 | $\tilde{\text{P}}$_O2sr_, $\tilde{\text{P}}$_O2sv_ | Parameters in the offset terms of the sympathetic neural activation equation | 30.00 | mmHg |  |
| 83 | k_sr_, k_sv_ |  | 2.00 | mmHg |  |
| 84 | g_sr_ |  | 90.00 | 1/(mmHg$\text{·}$min) |  |
| 85 | g_sv_ |  | 0.00 | 1/(mmHg$\text{·}$min) |  |
| 86 | $\text{θ}$_shn_ | Initial offset terms of the sympathetic neural activation equation | 2.16$\text{×}$10^2^ | 1/min |  |
| 87 | $\text{θ}$_srn_, $\text{θ}$_svn_ | Initial offset term of the sympathetic neural activation equation | 7.99$\text{×}$10^2^ | 1/min |  |
| 88 | f_es0_ | Parameters for the frequencies of spikes in the sympathetic efferent fibers | 9.67$\text{×}$10^2^ | spikes/min |  |
| 89 | f_esmax_ |  | 3.60$\text{×}$10^3^ | spikes/min |  |
| 90 | f_es∞_ |  | 1.26$\text{×}$10^2^ | spikes/min |  |
| 91 | w_csp_, w_csv_ |  | 5.00 | - |  |
| 92 | w_csh_ |  | 1.00 | - |  |
| 93 | w_psp_, w_psv_ |  | −0.34 | - |  |
| 94 | w_cv_ |  | 0.20 | - |  |
| 95 | w_pv_ |  | 0.10 | - |  |
| 96 | $\text{θ}$_v_ |  | −40.80 | spikes/min |  |
| 97 | f_esmin_ | Threshold of the sympathetic stimulation | 1.60$\text{×}$10^2^ | spikes/min |  |
| 98 | G_h_ | Gain of change in heart strength | 3.80$\text{×}$10^3^ | - |  |
| 99 | G_r_ | Gain of change in venous resistance | 1.04$\text{×}$10^2^ | mmHg$\text{·}$min$\text{/}$L |  |
| 100 | G_v_ | Gain of change in unstressed blood volume in the veins | −0.16 | L | Kurian et al. (2025a) |
| 101 | G_hs_ | Gain of change in heart rate | −2.20$\text{×}$10^−3^ | beats/min | Cheng et al. (2010) |
| 102 | G_hv_ |  | 1.50$\text{×}$10^−3^ | beats/min |  |
| 103 | $\text{k}_{\text{CO2}}$ | Parameters for local blood control | 1.43$\text{×}$10^3^ | mmHg | Kurian et al. (2025a) |
| 104 | $\text{g}_{\text{O2}}$ |  | 3.00 | mL/mL |  |
| 105 | $\text{τ}$_ac_ | Time constants of chemoreceptor response | 3.33$\text{×}$10^−2^ | min | Cheng et al. (2010) |
| 106 | $\text{τ}$_ap_ |  | 3.33$\text{×}$10^−2^ | min |  |
| 107 | $\text{τ}$_isc_ | Time constants of offset terms | 0.50 | min |  |
| 108 | $\text{τ}$_cc_ |  | 0.33 | min |  |
| 109 | $\text{τ}$_h_ | Time constant of change in heart strength | 0.13 | min |  |
| 110 | $\text{τ}$_r_ | Time constant of change in venous resistance | 0.10 | min |  |
| 111 | $\text{τ}$_v_ | Time constant of change in unstressed blood volume in the veins | 0.33 | min |  |
| 112 | $\text{τ}$_hs_ | Time constants of change in heart rate | 3.33$\text{×}$10^−2^ | min |  |
| 113 | $\text{τ}$_hv_ |  | 2.50$\text{×}$10^−2^ | min |  |
| 114 | $\text{C}_{\text{vO20}}$ | Nominal value of O_2_ concentration in the veins | 0.13 | mL/mL |  |
| 115 | $\text{τ}$_O2_ | Time constants of local blood flow control | 0.17 | min |  |
| 116 | $\text{τ}$_CO2_ |  | 0.33 | min |  |
| 117 | $\text{τ}_{\text{ventp}}$ | Time constants of ventilation regulation | 0.22 | min | Magosso and Ursino (2001) |
| 118 | $\text{τ}_{\text{ventc}}$ |  | 3.00 | min |  |

1. Effect of ketamine

| **P#** | **Parameter name** | **Description** | **Value** | **Unit** | **Source** |
| --- | --- | --- | --- | --- | --- |
| 119 | $\text{C}\text{L}_{\text{SK}}$ | *S*-ketamine elimination clearance | 1.78 | L/min | Kamp et al. (2020) |
| 120 | $\text{C}\text{L}_{\text{RK}}$ | *R*-ketamine elimination clearance | 1.58 | L/min |  |
| 121 | $\text{V}\text{1}_{\text{K}}$ | Central compartment volume for ketamine | 25.80 | L |  |
| 122 | $\text{Q}_{\text{K}}$ | Intercompartmental clearance for ketamine | 2.10 | L/min |  |
| 123 | $\text{V}\text{2}_{\text{K}}$ | Peripheral compartment volume for ketamine | 115.00 | L |  |
| 124 | $\text{V}\text{2}_{\text{NK}}$ | Peripheral compartment volume for norketamine | 240.00 | L |  |
| 125 | $\text{C}\text{L}_{\text{SNK}}$ | *S*-norketamine elimination clearance | 1.00 | L/min |  |
| 126 | $\text{Q}_{\text{SNK}}$ | *S*-norketamine intercompartmental clearance | 3.27 | L/min |  |
| 127 | MTT | Norketamine mean transit time | 26.60 | min |  |
| 128 | $\text{TR}_{\text{NK}}$ | Time constant of norketamine transit | 7.52×10^−2^ | 1/min |  |
| 129 | $\text{C}\text{L}_{\text{RNK}}$ | *R*-norketamine central clearance | 0.73 | L/min |  |
| 130 | $\text{Q}_{\text{RNK}}$ | *R*-norketamine intercompartmental clearance | 2.55 | L/min |  |
| 131 | $\text{C}_{\text{25,SK,CO}}$ | *S*-ketamine concentration for 25% change in CO | 4.00×10^−4^ | g/L | Kamp et al. (2021) |
| 132 | $\text{C}_{\text{25,SNK,CO}}$ | *S*-norketamine concentration for 25% change in CO | 1.60×10^−4^ | g/L |  |
| 133 | $\text{T}_{\text{1/2,SK,CO}}$ | *S*-ketamine effect compartment half-life for CO | 2.28 | min |  |
| 134 | $\text{T}_{\text{1/2,SNK,CO}}$ | *S*-norketamine effect compartment half-life for CO | 29.30 | min |  |
| 135 | $\text{Emax}_{\text{K,MAP}}$ | Maximum effect of ketamine on MAP | 51.60 | mmHg | Abuhelwa et al. (2022) |
| 136 | $\text{EC}_{\text{50,K,MAP}}$ | Ketamine concentration with half-maximal effect on MAP | 4.68×10^−4^ | g/L |  |
| 137 | $\text{n}_{\text{K,MAP}}$ | Exponent in the pharmacodynamic model of MAP | 2.04 | - |  |
| 138 | $\text{a}_{\text{10}}$ | Parameters in the equation coupling the effect of ketamine on MAP controller | −2.17×10^2^ | mmHg | Kurian et al. (2025b) |
| 139 | $\text{a}_{\text{11}}$ |  | 1.47 | - |  |
| 140 | $\text{a}_{\text{12}}$ |  | 14.01 | mmHg⋅min/L |  |
| 141 | $\text{a}_{\text{20}}$ | Parameters in the equation coupling the effect of ketamine on CO controller | −14.68 | L/min |  |
| 142 | $\text{a}_{\text{21}}$ |  | −5.28×10^−3^ | L/(min⋅mmHg) |  |
| 143 | $\text{a}_{\text{22}}$ |  | 3.02 | - |  |

CO: cardiac output; MAP: mean arterial pressure.

1. Respiratory control and mechanics

| **P#** | **Parameter name** | **Description** | **Value** | **Unit** | **Source** |
| --- | --- | --- | --- | --- | --- |
| 144 | G_cR_ | Gain of the central-chemoreceptor effects on RR | 0.06 | Breaths  /(min^2^$\text{·}$spikes) | Estimated |
| 145 | G_cP_ | Gain of the central-chemoreceptor effects on | -0.03 | mmHg  /(min$\text{·}$spikes) |  |
| 146 | $\text{τ}$_cR_ | Time constant of the central-chemoreceptor effects on RR | 0.12 | 1/min |  |
| 147 | $\text{τ}$_cP_ | Time constant of the central-chemoreceptor effects on P_mus,min_ | 0.12 | 1/min |  |
| 148 | G_pR_ | Gain of the peripheral-chemoreceptor effects on RR | 0.02 | Breaths  /(min^2^$\text{·}$spikes) |  |
| 149 | G_pP_ | Gain of the peripheral-chemoreceptor effects on P_mus,min_ | -0.01 | mmHg  /(min$\text{·}$spikes) |  |
| 150 | $\text{τ}$_pR_ | Time constant of the peripheral-chemoreceptor effects on RR | 0.13 | 1/min |  |
| 151 | $\text{τ}$_pP_ | Time constant of the peripheral-chemoreceptor effects on P_mus,min_ | 0.16 | 1/min |  |
| 152 | RR$\text{o}$ | Initial RR | 12.00 | Breaths/min | Albanese et al. (2016) |
| 153 | P_mus,mino_ | Initial P_mus,min_ | -3.68 | mmHg |  |
| 154 | R_mlo_ | Nominal value of the resistance from the mouth to the larynx | 0.01 | mmHg$\text{·}$min/L |  |
| 155 | R_lt_$\text{o}$ | Nominal value of the resistance from the larynx to the trachea | 4.10×10^−3^ | mmHg$\text{·}$min/L |  |
| 156 | R_tb_$\text{o}$ | Nominal value of the resistance from the trachea to the bronchi | 3.80×10^−3^ | mmHg$\text{·}$min/L |  |
| 157 | V_llu_ | Unstressed volume of the left lung | 0.57 | L |  |
| 158 | V_rul_ | Unstressed volume of the right lung | 0.69 | L |  |
| 159 | C_ll_ | Compliance of the left lung | 0.14 | L/mmHg |  |
| 160 | C_rl_ | Compliance of the right lung | 0.17 | L/mmHg |  |
| 161 | R_bll_ | Resistance from the bronchi to the left lung | 2.20×10^−3^ | mmHg$\text{·}$min/L |  |
| 162 | R_brl_ | Resistance from the bronchi to the right lung | 1.80×10^−3^ | mmHg$\text{·}$min/L |  |
| 163 | C_cw_ | Compliance of the chest wall | 0.33 | L/mmHg |  |

P_mus,min_, minimum respiratory muscle pressure; RR: respiratory rate.

**References**

Abuhelwa, A.Y., Somogyi, A.A., Loo, C.K., Glue, P., Barratt, D.T., and Foster, D.J. (2022). Population pharmacokinetics and pharmacodynamics of the therapeutic and adverse effects of ketamine in patients with treatment‐refractory depression. *Clin Pharm Ther* 112(3), 720–729. doi: 10.1002/cpt.2640.

Albanese, A., Cheng, L., Ursino, M., and Chbat, N.W. (2016). An integrated mathematical model of the human cardiopulmonary system: model development. *Am J Physiol Heart Circ Physiol* 310(7), H899–H921. doi: 10.1152/ajpheart.00230.2014.

Cheng, L., Ivanova, O., Fan, H.-H., and Khoo, M.C. (2010). An integrative model of respiratory and cardiovascular control in sleep-disordered breathing. *Respir Physiol Neurobiol* 174(1-2), 4–28. doi: 10.1016/j.resp.2010.06.001.

Guyton, A.C. (1980). *Arterial Pressure and Hypertension.* Philadelphia, PA: Saunders.

Kamp, J., Jonkman, K., van Velzen, M., Aarts, L., Niesters, M., Dahan, A., et al. (2020). Pharmacokinetics of ketamine and its major metabolites norketamine, hydroxynorketamine, and dehydronorketamine: a model-based analysis. *Br J Anaesth* 125(5), 750–761. doi: 10.1016/j.bja.2020.06.067.

Kamp, J., van Velzen, M., Aarts, L., Niesters, M., Dahan, A., and Olofsen, E. (2021). Stereoselective ketamine effect on cardiac output: a population pharmacokinetic/pharmacodynamic modelling study in healthy volunteers. *Br J Anaesth* 127(1), 23–31. doi: 10.1016/j.bja.2021.02.034.

Kurian, V., Jin, X., Nagaraja, S., Wallqvist, A., and Reifman, J. (2025a). A model to simulate human cardio-respiratory responses to different fluid resuscitation treatments after hemorrhagic injury. *Front Physiol* 16, 1613874. doi: 10.3389/fphys.2025.1613874.

Kurian, V., Jin, X., Wallqvist, A., Reifman, J., and Nagaraja, S. (2025b). A model to simulate human cardio-respiratory responses to ketamine analgesia. *Under Review*.

Magosso, E., and Ursino, M. (2001). A mathematical model of CO_2_ effect on cardiovascular regulation. *Am J Physiol Heart Circ Physiol* 281(5), H2036–H2052. doi: 10.1152/ajpheart.2001.281.5.H2036.

Mazzoni, M.C., Borgstrom, P., Arfors, K.-E., and Intaglietta, M. (1988). Dynamic fluid redistribution in hyperosmotic resuscitation of hypovolemic hemorrhage. *Am J Physiol Heart Circ Physiol* 255(3), H629–H637. doi: 10.1152/ajpheart.1988.255.3.H629.

Ursino, M., Magosso, E., and Avanzolini, G. (2001). An integrated model of the human ventilatory control system: the response to hypercapnia. *Clin Physiol* 21(4), 447–464. doi: 10.1046/j.1365-2281.2001.00349.x.

Table S2. List of the two most sensitive parameters for MV and RR at five different airway obstruction levels.

| Obstruction Level (%) | Rank | MV | | RR | |
| --- | --- | --- | --- | --- | --- |
|  |  | Parameter | Sensitivity | Parameter | Sensitivity |
| 0 | 1 | P_mus,mino_ | 0.28 | RR_o_ | 0.74 |
|  | 2 | RR_o_ | 0.26 | P_mus,mino_ | -0.28 |
| 25 | 1 | P_mus,mino_ | 0.27 | RR_o_ | 0.74 |
|  | 2 | RR_o_ | 0.24 | P_mus,mino_ | -0.29 |
| 50 | 1 | P_mus,mino_ | 0.26 | RR_o_ | 0.74 |
|  | 2 | V_V0_ | 0.23 | P_mus,mino_ | -0.30 |
| 75 | 1 | R_mlo_ | 0.34 | RR_o_ | 0.64 |
|  | 2 | V_V0_ | 0.20 | R_mlo_ | 0.42 |
| 100 | 1 | HS_0_ | 5.07 | G_pR_ | 0.63 |
|  | 2 | K_SR_ | 5.07 | f_acmax_ | 0.63 |

f_acmax_, upper saturation level of frequency discharge in the afferent chemoreceptor; G_pR_, gain of the peripheral-chemoreceptor effects on respiratory rate; HS_0_, initial heart strength; K_SR_, gain of the sympathetic effects on initial arterial resistance; MV, minute ventilation; P_mus,mino_, initial minimum respiratory muscle pressure; R_mlo_, nominal value of the resistance from the mouth to the larynx; RR, respiratory rate; RR_o_, initial respiratory rate; V_V0_, initial blood volume in the veins.

Figure S1. Model validation using data from the normal ventilation and hyperventilation scenarios in *Study 8*. Experimental (filled circles) and predicted (solid lines) (**A,B**) end-tidal carbon dioxide (ETCO_2_), (**C,D**) mean arterial pressure (MAP), and (**E,F**) heart rate (HR). The error bars denote two standard errors of the mean. The timelines at the top of panels **A** and **B** illustrate the experimental scenario, where the subjects underwent hemorrhage (25% of blood volume) followed by ventilation changes. SV indicates that the subjects were on spontaneous ventilation during that period. MV: minute ventilation; RMSE: root mean square error.
